# Host tRNA-Derived RNAs Target the 3′Untranslated Region of SARS-CoV-2

**DOI:** 10.3390/pathogens11121479

**Published:** 2022-12-06

**Authors:** Emily N. Hendrickson, Marna E. Ericson, Lynne T. Bemis

**Affiliations:** 1 Department of Biomedical Sciences, Duluth Campus, Medical School, University of Minnesota, Duluth, MN 55812, USA; 2 The Hormel Institute, University of Minnesota, Austin, MN 55912, USA; 3 T Lab Inc., 910 Clopper Road, Suite 220S, Gaithersburg, MD 20878, USA

**Keywords:** tRNA fragments, tRF, tRNA-derived RNAs, tDR, small noncoding RNA, host-viral interactions, SARS-CoV-2, Variants of Concern, VOCs

## Abstract

The COVID-19 pandemic revealed a need for new understanding of the mechanisms regulating host–pathogen interactions during viral infection. Transfer RNA-derived RNAs (tDRs), previously called transfer RNA fragments (tRFs), have recently emerged as potential regulators of viral pathogenesis. Many predictive studies using bioinformatic approaches have been conducted providing a repertoire of potential small RNA candidates for further analyses; however, few targets have been validated to directly bind to SARS-CoV-2 sequences. In this study, we used available data sets to identify host tDR expression altered in response to SARS-CoV-2 infection. RNA-interaction-prediction tools were used to identify sequences in the SARS-CoV-2 genome where tDRs could potentially bind. We then developed luciferase assays to confirm direct regulation through a predicted region of SARS-CoV-2 by tDRs. We found that two tDRs were downregulated in both clinical and in vitro cell culture studies of SARS-CoV-2 infection. Binding sites for these two tDRs were present in the 3′ untranslated region (3′UTR) of the SARS-CoV-2 reference virus and both sites were altered in Variants of Concern (VOCs) that emerged later in the pandemic. These studies directly confirm the binding of human tDRs to a specific region of the 3′UTR of SARS-CoV-2 providing evidence for a novel mechanism for host–pathogen regulation.

## 1. Introduction

In late 2019, a novel zoonotic virus, a beta coronavirus now known as SARS-CoV-2, was identified in Wuhan, China [1,2,3]. The disease caused by the virus, referred to as coronavirus disease 2019 (COVID-19), was classified as a pandemic, and new variants of SARS-CoV-2 began to emerge [4]. Each variant that posed a significant risk to public health was termed a variant of concern (VOC) by the World Health Organization. These VOCs impact the need for changes in vaccines and testing protocols and pose challenges to the development of novel therapeutic interventions [5,6,7]. The persistence of new RNA virus outbreaks like SARS-CoV-2 coupled with the possibility of an increase in frequency of new epidemics highlights a heightened need for novel antiviral therapeutic targets [8]. The function of small noncoding RNAs in response to viral infection are largely untested and present a prospective therapeutic target to alleviate the burden of viral diseases.

Small noncoding RNAs (sncRNAs) are short RNA molecules (<100 nt) that have been implicated in regulating a variety of context-dependent cellular processes [9]. Two well-known families of sncRNAs, microRNAs (miRNAs) and transfer RNA-derived RNAs (tDRs), are involved in the regulation of gene expression and are associated with cancer and viral infections [10,11]. tDRs are derived from multiple regions of tRNAs by a directed process and their abundance has resulted in a confusing array of overlapping names: tRNA-halves, tsRNAs, tiRNAs and tRNA fragments, etc. Recently a new naming system has been proposed for this class of small noncoding RNAs, http://trna.ucsc.edu/tDRnamer/docs (accessed on 12 November 2022). While small noncoding RNAs with antiviral activity are a well-defined phenomenon in plants and invertebrates, only recently have studies implicated such noncoding RNAs in the antiviral response in mammals [12,13,14]. Small noncoding RNAs, including certain miRNAs and tDRs, show altered expression during viral infection [15]. Viruses are also known to produce sncRNAs from their own genomes [16]. Viral encoded small RNAs have recently been identified in SARS-CoV-2-infected cells [17,18].

The expression of a large number of microRNAs, produced by both host and virus, have been identified as altered in SARS-CoV-2 infected cells, and yet limited knowledge of their function exists [19,20]. In addition, the role of tDRs in SARS-CoV-2 infection remains essentially unexplored. Furthermore, a host tDR has been shown to contribute to the anti-viral response in respiratory syncytial virus [21,22]. Many publicly available next generation sequencing (NGS) studies, focus on the miRNAs altered in response to SARS-CoV-2 infection; however, the tDRs, have been characterized in only a few of those studies [17,23,24].

In this study we accessed available RNA sequencing data to identify tDRs that are differentially expressed in the plasma of patients with severe COVID-19 compared to healthy controls [25]. Many studies show that tDRs are frequently found in accessible patient samples, including plasma and serum, and have been proposed as potential biomarkers of disease [11]. We then analyzed another available dataset from an in vitro model of SARS-CoV-2-infected Calu-3 cells [26]. We chose Calu-3 cells because they are human airway epithelial cells, the site of SARS-CoV-2 infection. Our goal was to identify the altered expression of human tDRs during SARS-CoV-2 infection that were consistent across the two data sets. The RNA seq data that we analyzed was originally collected to examine miRNA expression in patients diagnosed with COVID-19; however, the methods utilized for NGS also often allow the collection and detection of tDRs. Two human tDRs were identified as altered in response to infection by SARS-CoV-2: one derived from the 5′ end of tRNA-Gly (30 base pairs) and the other from the 5′ end of tRNA-Val (31 base pairs). We hypothesized that if a binding site in the SARS-CoV-2 genome was important for anti-viral regulation by the host, it might have been altered in one of the SARS-CoV-2 variants of concern (VOCs), Alpha (B.1.1.7), Delta (B.1.617.2) or Omicron (B.1.1.529) subvariants BA.1 and BA.2. We identified one predicted binding site in the SARS-CoV-2 reference sequence that had been altered in two VOCs resulting in a reduced binding affinity for the host tDRs. We developed luciferase assays to evaluate regulation through the predicted binding site from the viral reference sequence and the altered sequences from two VOCs, Delta and BA.2. We also developed tDR sponges to soak up the specific host tDRs thereby reducing regulation through the predicted binding site from SARS-CoV-2. This study establishes that two tDRs produced by the human host can bind to sequences in the 3′Untranslated Region (3′UTR) of SARS-CoV-2 and identifies two SARS-CoV-2 VOCs that show reduced binding. The expression of the SARS-CoV-2 binding sites in luciferase plasmids confirmed that the predicted tDR binding sites are targeted by human tDRs. These studies show the direct binding of human tDRs to sequences in the 3′UTR of SARS-CoV-2, identifying a novel regulatory mechanism for this virus.

## 2. Results

### 2.1. tDR Expression Is Altered in SARS-CoV-2 Infected Patients’ Plasma Compared to Uninfected Control Plasma

**Figure 1:**
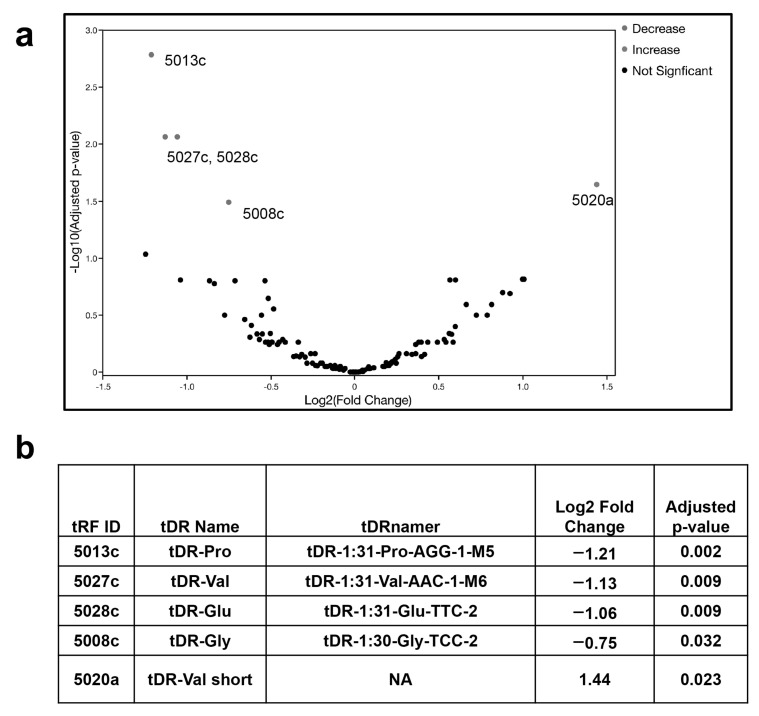
Altered expression of a subset of human tDRs in plasma from SARS-CoV-2-infected patients compared to plasma samples from uninfected individuals. (**a**) Volcano plot showing the differential expression of tDRs in SARS-CoV-2-infected patients. The significantly altered tDRs are indicated by color with red being increased and blue designating the decreased tDRs, the *p*-value cut-off is <0.05. Log2 fold change, and *p*-value are those corresponding to the volcano plot. (**b**) Table of tDRs significantly altered in SARS-CoV-2-infected patients compared to uninfected individuals with the naming conventions: tRF ID is from the original data base used in the analysis and using the former name of tRNA fragments (http://genome.bioch.virginia.edu/trfdb/ [27,28] accessed on 17 January 2022); the tDR name is a shortened name used for convenience in this manuscript; the tDRnamer is the most accurate naming convention for the tRNA-derived RNAs [29,30], http://trna.ucsc.edu/tDRnamer/docs/ accessed on 12 November 2022; NA is not applicable for 5020a because it is too short to be identified in the tDRnamer data base (http://trna.ucsc.edu/tDRnamer/index.html) accessed on 12 November 2022.

We utilized an available data set to identify tDRs in plasma samples from patients with a confirmed infection of SARS-CoV-2 compared to those without infection [25]. These data had previously been examined for the altered expression of host miRNAs; however, next generation sequencing libraries designed to identify small RNAs often contain tDRs, which are excluded when analyzing the data for miRNAs [25]. The data set is included in the Bioproject PRJNA702830 and consists of 19 samples from patients with confirmed SARS-CoV-2 infection and 10 samples from uninfected controls. These small RNA-seq libraries were compared to the tDR database, which is available online at http://genome.bioch.virginia.edu/trfdb/ (accessed on 17 January 2022) (Figure 1). 

Of the 124 tDRs analyzed, five were significantly differentially expressed (*p* < 0.05) in patients diagnosed with COVID-19 compared to uninfected individuals (Figure 1). The 5′-tDRs 5008c, 5013c, 5027c and 5028c were decreased in COVID-19 patients, and 5020a was increased. As previously reported, the 5′tDRs, were significantly changed in response to SARS-CoV-2 infection [17]. In addition, an assessment of patients above 60 years old that were diagnosed with COVID-19 compared to patients below 60 years old with COVID-19 revealed that 5013c was increased and 5019a was decreased (File S1). When COVID-19 patients above 60 years old were compared to healthy patients above 60 years old, thirteen 5′ tDRs and two 3′ tDRs were significantly altered (File S2). Comparisons between the genders revealed four tDRs that were significantly differentially expressed: 3022a and 3022b were decreased in uninfected females compared to uninfected males (File S3, Table S3a); 3005a and 5013c were both decreased in males diagnosed with COVID-19 compared to uninfected males (File S3, Table S3b). No tDRs from this data set were significantly altered in females diagnosed with COVID-19 compared to males diagnosed with COVID-19.

### 2.2. Identification of Altered tDR Expression in SARS-CoV-2-Infected Calu-3 Lung Cells

To refine the search for human tDRs of interest, we analyzed another available data set. The second data set was originally established to identify small noncoding RNA using small RNA-seq in Calu-3 cells infected with SARS-CoV-2 compared to mock infected cells. These data were accessed at NCBI (GEO: GSE148729). In the original analysis, these data were examined for altered expression of the host miRNAs [26]. Using the same methods employed with the plasma samples (Figure 1), we analyzed the sequence data from small RNA-seq libraries of infected and mock infected Calu-3 cells and compared them to the tDR database (http://genome.bioch.virginia.edu/trfdb/ accessed on 17 January 2022).

We found that the expression of several of the same human tDRs from the plasma samples was also significantly altered in response to direct infection by SARS-CoV-2 (Table 1). Two tDRs, 5027c and 5008c, were significantly decreased in the treated Calu-3 cells at the 24-hour timepoint compared to the mock-treated cells. Three tDRs, 5027c, 5008c and 5020a were altered in the same direction as they were in the previously analyzed plasma samples. The expression of 5028c was increased in the SARS-CoV-2-infected cells; however, 5028c exhibited decreased expression in the plasma of SARS-CoV-2-infected patients. No detectable expression of 5013c was observed in the Calu-3 cells.

### 2.3. RNA Hybridization Analysis to Detect Potential Binding Sites in SARS-CoV-2 for Selected tDRs

**Figure 2:**
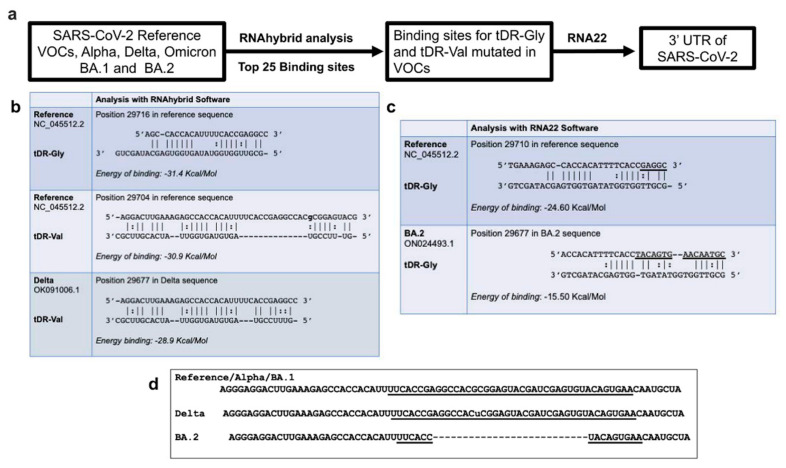
Predicted binding of tDR-Gly and tDR-Val to the SARS-CoV-2 genome. (**a**) A schematic of the process used to identify potential hybridization sites for tDRs in the SARS-CoV-2 genomic sequences. RNAhybrid was used to predict the top 25 binding sites for tDR-Gly and tDR-Val in the SARS-CoV-2 reference sequence (NC_045512.2), and in the VOCs, Alpha (OV054768.1), Delta (OK091006.1), Omicron BA.1 (OL672836.1) and BA.2 (ON024493.1). The hybridization prediction software, RNA22, which uses a different algorithm than RNAhybrid was also used to test each tDR against the indicated sequences. The application of these two methods led to the choice of a site in the 3′UTR of SARS-CoV-2, for further analysis. (**b**) RNAhybrid predictions for tDR-Gly and tDR-Val binding to the 3′ end of SARS-CoV-2 showed a minimal free energy of binding similar to that expected for a microRNA and its target [33]. The sequence in this region of the Delta VOC contains a single nucleotide polymorphism changing a **G** in the reference sequence to a **U** in Delta. The alteration in the Delta VOC changes the predicted binding shown for tDR-Val to the Delta VOC. (**c**) The RNA22 prediction software for the 3′UTR of SARS-CoV-2 showed that tDR-Gly but not tDR-Val was predicted to bind, and binding was predicted to be altered in the BA.2 sequence. The sites predicted by RNAhybrid and RNA22 are slightly different due to the numbering conventions and parameters for each software package. (**d**) Sequences in the region of SARS-CoV-2 predicted to bind to tDR-Gly or Val are not altered in the Alpha or Omicron BA.1 variants. A base-pair change is detected in this region in the Delta VOC (u) and a 26-base-pair deletion is observed in the BA.2 variant. The underlined bases in the Reference/Alpha/Omicron BA.1 sequences designate the stem loop II (s2m) region of SARS-CoV-2 [34,35], which is partially deleted in BA.2.

The choice of specific tDRs for further experimentation was based on those tDRs identified in both the patient plasma and Calu-3 cell line NGS data as significantly decreased in expression in response to infection [25,26]. Thus, we focused the next studies on the two tDRs that were downregulated in both data sets, 5027c and 5008c (Figure 2, Table 1). The early studies identifying tDRs utilized many different naming conventions including tRF-ID numbers such as 5027c and 5008c, thus we searched the tDRnamer website using the sequences for 5027c and 5008c to identify their tDR names (File S4). For the following experiments, we have abbreviated the names of these two tDRs to tDR-Gly and tDR-Val to comply with the new method of naming tRNA-derived fragments (http://trna.ucsc.edu/tDRnamer/docs/ accessed 12 November 2022).

Using the sequences for tDR-Gly (5′-GCGTTGGTGGTATAGTGGTGAGCATAGCTG) and for tDR-Val (5′-GTTTCCGTAGTGTAGTGGTTATCACGTTCGC), we examined the predicted RNA binding of these two tDRs to SARS-CoV-2 and the variants of concern (VOCs) Alpha, Delta, Omicron subvariants BA.1, and BA.2, by using RNAhybrid [31] followed by analysis with RNA22 software [32] (Figure 2).

tDR-Gly and tDR-Val are predicted to hybridize in multiple regions across the panel of SARS-CoV-2 genomes tested (File S3). To narrow down a region of regulation for further study, we hypothesized that if the binding of tDRs plays an anti-viral role in SARS-CoV-2 regulation, we would expect that one or more VOCs would have an alteration in the predicted hybridization stability represented by the minimal free energy of binding. A single-point mutation in the Delta variant showed an altered binding energy (Figure 2b) and the BA.2 variant was also significantly altered in this region with 26 base pairs deleted resulting in the loss of the predicted binding site for tDR-Val. The 3′UTR region of the SARS-CoV-2 genome matched our criteria of having mutations in the VOCs that decrease the predicted binding of tDRs, this region includes the structural element called stem loop II (s2m). Stem loop II is a previously characterized structure found in many RNA viruses [34,35]. The alterations in the VOC sequences examined for potential binding are shown in Figure 2d.

The predicted binding site for tDR-Gly is not altered in the Delta variant because the **G** to U change is outside of the predicted binding site, while the binding of tDR-Val is expected to be reduced (Figure 2b). The RNA22 software predicted the binding of tDR-Gly in the reference sequence and altered the binding in BA.2 (Figure 2c). However, the RNA22 software did not predict the binding of tDR-Val in this specific region of the SARS-CoV-2 reference sequence or in any of the VOCs examined in this study.

### 2.4. The Predicted tDR Binding Sequences from the SARS-CoV-2 Reference Genome and VOCs Were Placed Downstream of Luciferase to Determine if Host Factors Could Regulate Expression 

**Figure 3:**
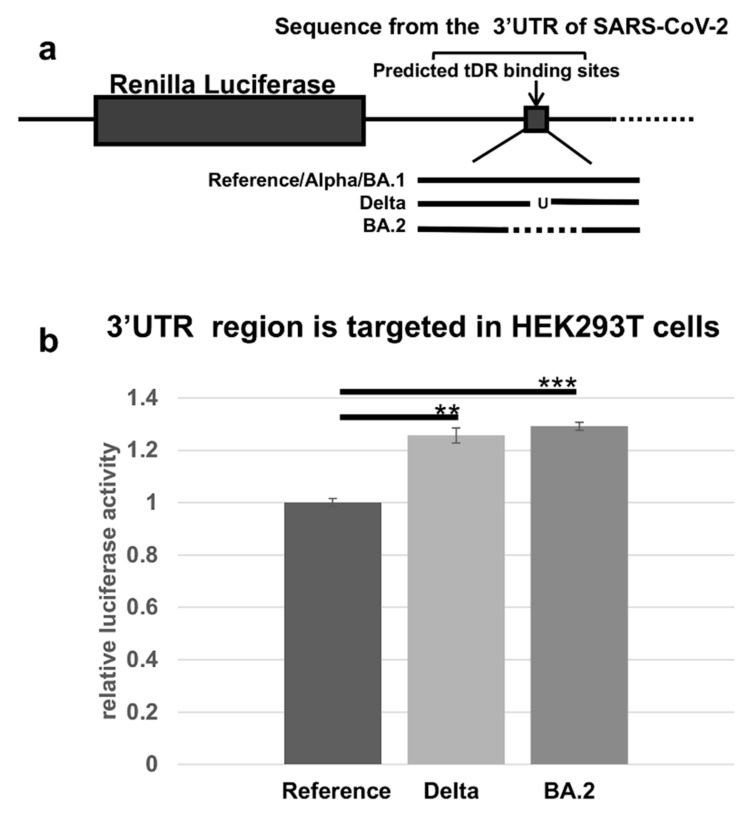
Direct regulation through the 3′UTR of SARS-CoV-2 as detected by the loss of luciferase activity. (**a**) A schematic showing the luciferase constructs in the psi-Check-2 plasmid; the reference sequence is the same in the VOCs Alpha and Omicron BA.1 in this region; Delta is altered by one base and the sequence for BA.2 is the region of the 3′UTR encompassing a 26-base-pair deletion. (**b**) Human embryonic kidney cells (HEK293T) were transfected with the luciferase constructs. The transfections were performed in triplicate. The luciferase activity of both VOCs was compared to the reference sequence from SARS-CoV-2 as an unmutated control. The alterations in the sequence resulted in significant changes in the luciferase activity in Delta (** *p*-value < 0.0005) or in BA.2 (*** *p*-value < 0.00005). The transfections were performed in triplicate. The results were compared to the reference sequence as a percent of control and the error bars refer to ±standard deviation.

To test if this part of the 3′UTR of SARS-CoV-2 is directly regulated by a host response, we designed and constructed luciferase reporter plasmids expressing the predicted binding site for the SARS-CoV-2 reference sequence and from the Delta and BA.2 variants. Our hypothesis was that the alterations in the 3′UTR of the Delta or BA.2 variants would reduce the regulation of SARS-CoV-2 through this region based on their predicted minimal free energy of binding. The regulation through each of these predicted binding sites was tested using luciferase activity assays such as those developed previously for miRNA studies (Figure 3) [36].

The expression of luciferase in the HEK293T cells, transfected with the constructs containing sequences from Delta or BA.2, was significantly increased compared to the reference sequence. These findings indicated the VOCs are less susceptible to downregulation by the host compared to the reference sequence.

### 2.5. Sponge Constructs of tDR-Val and tDR-Gly Were Co-Transfected with the Psi-Reference and VOC Plasmids to Determine Direct Regulation by tDR-Gly and tDR-Val

**Figure 4:**
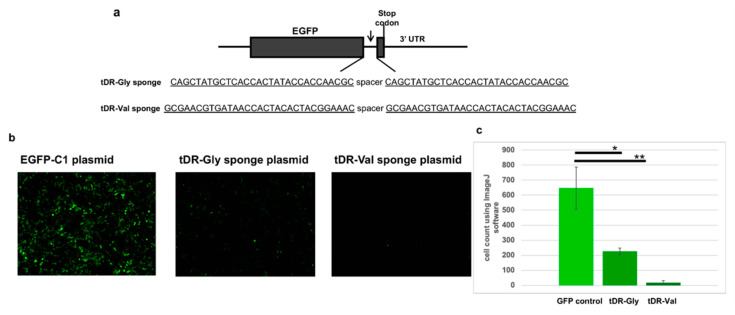
Plasmids with the anti-sense sponge sequences for tDR-Gly and tDR-Val were transfected into the HEK293T cells. (**a**) A schematic showing the sponge constructs in the EGFP-C1 plasmid. Two copies of the anti-sense sequence for tDR-Gly and tDR-Val were inserted at the 3′ end of the EGFP protein-encoding sequence. The anti-sense sequences for tDR-Gly and tDR-Val are underlined. (**b**) HEK293T cells were transfected with either the empty vector EGFP-C1 plasmid, tDR-Gly-EGFP-C1 sponge (tDR-Gly) or the tDR-Val-EGFP-C1 sponge (tDR-Val). (**c**) ImageJ software [38] was used to count the green cells in triplicate wells. The results were compared to the EGFP-C1 plasmid (GFP control). Error bars refer to ±standard deviation (* *p*-value < 0.05, ** *p*-value < 0.005).

Reagents for inhibiting tDRs are not readily available commercially. However, these small RNAs may be targeted by inhibitors similar to those used to inhibit microRNAs. In early studies of microRNAs, sponge constructs were described that soak up the microRNAs [37]. In this study, we developed sponge constructs in GFP-expressing plasmids to decrease the concentration of the available host tDRs, tDR-Gly and tDR-Val (Figure 4). 

The transfection of the HEK293T cells with the GFP expressing plasmid, EGFP-C1, revealed numerous green-expressing cells after 24 hours of transfection (Figure 4b). However, the cells transfected with plasmids expressing the sponge sequences for either tDR-Gly (antisense to tDR-Gly) or tDR-Val (antisense to tDR-Val) showed a reduction in GFP expression (Figure 4). The quantification of the green cells in triplicate wells was accomplished using the ImageJ software program and showed a significant reduction in GFP expression (Figure 4c). The reduction in GFP indicates that the sponge constructs are able to bind to the host tDRs.

The HEK293T cells were then co-transfected with the EGFP-C1 sponge plasmids or an empty vector control, as well as the psi-Check2 plasmids containing the 3′UTR sequences from the reference, Delta or BA.2, previously described in Figure 3.

**Figure 5:**
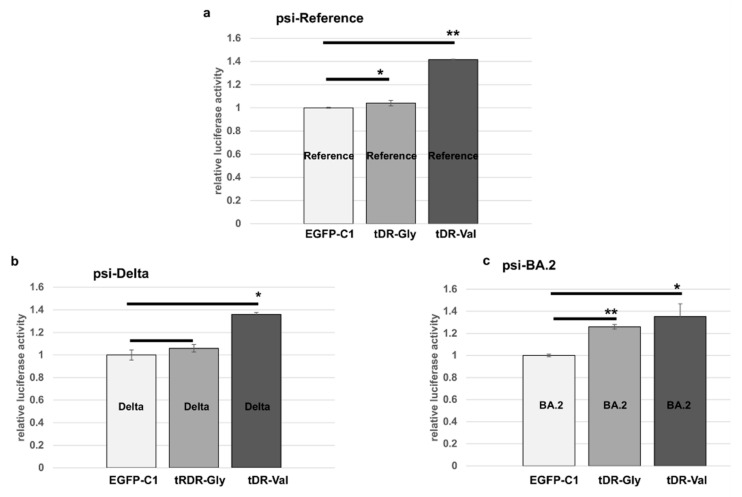
Sponges specific to tDR-Gly and tDR-Val block the targeting of the 3′UTR sequences from SARS-CoV-2. (**a**) HEK293T cells were transfected with the psi-reference sequence and either the EGFP-C1 empty vector control (EGFP-C1), tDR-Gly EGFP-C1 sponge (tDR-Gly) or the tDR-Val EGFP-C1 sponge (tDR-Val). (**b**) HEK293T cells transfected as in (**a**) but with the psi-Delta construct. (**c**) HEK293T cells transfected as in (**a**) but with the psi-BA.2 construct. The transfections were performed in triplicate. The results were compared to the EGFP-C1 empty vector (control) as a percent of control and the error bars refer to ±standard deviation (* *p*-value ≤ 0.05, ** *p*-value < 0.005).

The tDR-Val sponge blocked the downregulation of luciferase in the reference and both variants, while the tDR-Gly sponge impacted the luciferase activity in the cells transfected with the psi-BA.2 construct and to a lesser degree in the psi-reference-containing cells. In summary, these data show that the sequences within the 3′UTR of SARS-CoV-2 are targetable by the host tDR-Val and tDR-Gly (Figure 5). However, the single nucleotide change in the Delta sequence does not significantly alter its regulation by tDR-Gly compared to the reference sequence. The direct testing of these sequences in luciferase vectors is consistent with the binding predictions shown in Figure 2.

## 3. Discussion

The discovery of novel viruses like SARS-CoV-2 emphasizes the necessity of developing a better understanding of the cellular response to viral infection. The increased understanding of viral and host interactions will aid in the development of new therapeutic interventions. Noncoding RNAs, including tDRs, have been proposed as future targets for therapeutic intervention [39]. However, few studies have focused on defining the expression or function of tDRs during SARS-CoV-2 infection. We identified tDRs with altered expression in samples from COVID-19 patients and fine-tuned the scope of our studies with data from SARS-CoV-2 treated human lung cells (Table 1). We then identified the most likely binding sites for two downregulated human tDRs in the SARS-CoV-2 genome using hybridization prediction software [31,32]. The two tDRs, tDR-Gly and tDR-Val, are derived from the 5′ end of their respective cognate tRNAs. Many sites of potential binding of these two human tDRs to SARS-CoV-2 genomic sequences were identified (File S5). To narrow down a specific binding site for study, we examined VOCs to identify those altering the predicted hybridization site for either tDR-Gly or tDR-Val. Our hypothesis was that since tDR-Gly and tDR-Val are downregulated in response to SARS-CoV-2 infection (Figure 1, Table 1), they might play a role in the host response meant to defend against the viral pathogen. Other known host defense responses have been shown to be downregulated following infection by SARS-CoV-2 [40] and include the downregulation of DICER [41], an enzyme also known to generate tDRs [11]. 

The regulation by tDRs of SARS-CoV-2 could be part of an RNA interference (RNAi) response by the host in SARS-CoV-2-infected cells. The concept that RNAi is part of the immune response in mammals has been controversial, even though it is well documented as part of the anti-viral response in plants and insects [12]. RNAi requires the function of DICER-like enzymes that cleave the RNA viral genome following targeting by a small RNA [42]. The RNAi response is downregulated in patients infected with SARS-CoV-2; however, the significance of this finding in terms of the immune response was not appreciated because of the suspected absence of an anti-viral DICER. Recently an anti-viral DICER was discovered in mammalian cells, opening the way to a better understanding of this pathway in the mammalian anti-viral response [43]. Our finding that specific tDRs, tDR-Gly and tDR-Val, are downregulated in SARS-CoV-2- infected cells adds support to the possibility of RNAi regulation of this virus.

Small noncoding RNAs have previously been studied in SARS-CoV-2-infected patient samples and cell lines [44]. However, to our knowledge direct targeting of the virus by tDRs has not been previously reported. In this study, we used predictions of RNA hybridization to detect the regions of SARS-CoV-2 that might bind tDR-Gly and tDR-Val. The region that was most significantly altered included the part of the 3′UTR of the SARS-CoV-2 virus that contains the s2m structure. The s2m structure is a genomic structure highly conserved in several viral families and thought to be evolutionarily conserved in the SARS-related coronaviruses [35]. It has been hypothesized to be part of an unidentified host-RNAi response [45]. We developed luciferase assays, which included the s2m region and the sequence variations from Delta and BA.2, and we confirmed that human cells possess the needed cellular components to downregulate expression through this region of the SARS-CoV-2 genome (Figure 3). 

To study the direct regulation of this region of SARS-CoV-2 by the two tDRs, we developed sponge constructs to soak up the specific tDRs (Figure 4). Sponges containing the anti-sense sequence of the regulatory small RNA have previously been reported to soak up microRNAs [37,46]. In this report, we show that sponges for tDR-Gly and tDR-Val expressed from a GFP plasmid are able to block the expression of GFP. Furthermore, by expressing both the sponge and SARS-CoV-2 target sequences together in human cells, we were able to show that tDR-Gly and tDR-Val are required for downregulation through this portion of the 3′UTR of the SARS-CoV-2 virus. Our studies show that the 3′UTR of SARS-CoV-2 has a binding site targeted by tDRs and that tDR-Val and tDR-Gly have reduced binding in the Omicron BA.2 variant. Our studies also show that tDR-Val binding is reduced in the Delta VOC. A limitation of the study is that the proteins involved in this regulatory mechanism have not been confirmed. Future studies, to further our understanding of the regulation of SARS-CoV-2 by tDRs will require using the intact virus. New understanding of this novel mechanism of regulation will better inform our understanding of the host response to SARS-CoV-2 and potentially to other viral pathogens.

## 4. Materials and Methods

### 4.1. Data Analysis of Small RNAs in Patients’ Plasma

All 3′ and 5′ human tDR sequences were accessed from tRFdb (http://genome.bioch.virginia.edu/trfdb/ accessed 17 January 2022). The unique sequences were then assembled into a single annotated reference sequence in Geneious Prime 2022.0.2 (Biomatters Ltd., Auckland, New Zealand). The full SRA dataset was downloaded from NCBI (Bioproject PRJNA702830) as fastq files. The adapter sequences were right end trimmed using the BBDuk Geneious Prime plugin (https://sourceforge.net/projects/bbmap/ accessed 21 March 2022) (k = 12, trimq = 13, minlength = 12 and all other default parameters). Reads were evaluated for quality before and after trimming using FastQC v. 0.11.9 [47] (FastQC: A Quality Control Tool for High Throughput Sequence Data [Online] accessed 21 March 2022) before and after trimming. Trimmed reads were mapped to the reference sequence using the Bowtie Geneious Prime plugin [48]. Read counts were quantified then normalized and compared using the DESeq2 Geneious Prime plug in [49]. An adjusted *p*-value of *p* < 0.05 was used to determine the differentially expressed tDRs.

### 4.2. Data Analysis of Small RNA Libraries from Calu-3 Lung Cells Infected with SARS-CoV-2

The full SRA dataset was downloaded from NCBI (Bioproject PRJNA702830) as fastq files. The sequences were right end trimmed in two passes using the BBDuk Geneious Prime plugin (https://sourceforge.net/projects/bbmap/ accessed 21 March 2022). For the first pass, the Illumina adapters were trimmed using the parameters k = 12, trimq = 13, minlength = 12 and all other default parameters. In the second pass, the poly-A tails were trimmed. All further analysis was completed as described above.

### 4.3. RNAhybrid and RNA22 Analysis

The tDRs, 5008c and 5027c (now called tDR-Gly and tDR-Val), were used to search the full sequence of SARS-CoV-2 (NC_045512.2) for hybridization using RNAhybrid version 2.2 [31]. Once the binding sites for the reference sequence, were confirmed, a sequence corresponding to each of the VOCs, Alpha (OV054768.1), Delta (OK091006.1), Omicron BA.1 (OL672836.1) and BA.2 (ON024493.1), was selected from NCBI virus to determine if the same binding sites existed in the VOCs. RNAhybrid was used to detect any changes in the minimal free energy of binding (mfe) from that of the reference sequence. We used the default settings in RNAhybrid with the number of hits per target being 25 and the approximate *p*-value set for 3utr_human. The BA.2 sequence was also checked at 50 hits per target because it did not show a significant hit in the region of interest at the 25 hits per target setting. Sequences were also examined in the program RNA22 for significant binding in the region of the s2m in the reference sequence and the VOCs. RNA22 was utilized from the website with no changes in the parameters [32]. tDR 5008c (now tDR-Gly) was detected as binding to SARS-CoV-2 in this region by RNA22 in the reference sequence and all variants. The sequences of the tDRs used to search RNA22 were tDR-Gly 5′GCGUUGGUGGUAUAGUGGUGAGCAUAGCUG and tDR-Val GUUUCCGUAGUGUAGUGGUUAUCACGUUCGC. 

### 4.4. Luciferase Plasmids Were Constructed Using a Region of the 3′UTR from the Index Virus Reference Sequence, Delta VOC and the Omicron BA.2 VOC

A region of the 3′-UTR of SARS-CoV-2 from the reference sequence, including predicted binding sites for tDR-Gly and tDR-Val, was cloned into the psiCheck 2 dual luciferase vector using XhoI and NotI restriction enzymes and the sequence was confirmed by submission for sequencing to ACGT Inc. (Wheeling, IL, USA). The sequence used from SARS-CoV-2 for psi-reference is 5′AGGGAGGACTTGAAAGAGCCACCACATTTTCACCGAGGCCACGCGGAGTACGATCGAGTGTACAGTGAACAATGCTA. In addition, the same region with either the one base-pair change observed in the Delta variant (psi-Delta) 5′ AGGGAGGACTTGAAAGAGCCACCACATTTTCACCGAGGCCACTCGGAGTACGATCGAGTGTACAGTGAACAATGCTA or the region missing 26 base pairs (psi-BA.2) 5′AGGGAGGACTTGAAAGAGCCACCACATTTTCACCTACAGTGAACAATGCTA was cloned into psiCheck 2 at the Xho1 and NotI restriction sites using the methods previously described [50]. These plasmids were transfected into the HEK293T cells. For the transfection 50,000 cells per well were seeded 24–36 h prior to the transfection in 24-well plates and transfected with Lipofectamine 2000 (Invitrogen, Carlsbad, CA, USA) according to the manufacturer’s instructions. Following 24–48 h of transfection, luciferase assays were conducted using the dual luciferase reporter kit (Promega, Madison, WI, USA) following the methods provided by the manufacturer. 

### 4.5. Sponge Constructs Were Designed to Bind Cellular tDR-Gly and tDR-Val to Inhibit Their Function

The EGFP sponge plasmids were constructed by placing two antisense sequences with a spacer in between (shown in Figure 4) and cloning the sequences into the EGFP-C1 plasmid at the XhoI and EcoRI restriction sites. Plasmids were sequence-confirmed by submission to ACGT Inc, as described above. The co-transfection of EGFP-C1 controls and EGFP-C1 sponge plasmids with psi-Check 2-derived plasmids was conducted using Lipofectamine 2000 as previously described [36,50]. Images were captured 24 h following transfection using the EVOS M7000 inverted wide-field epifluorescence microscope (thermofisher.com accessed on 29 August 2022). The quantification of cells was conducted on triplicate wells using ImageJ software [38]. The HEK293T cells used for these studies were recently authenticated (Labcorp, Burlington, NC, USA). 

Statistical analysis for transfection studies: All the data are expressed as a percent of the control with standard deviations. The Student’s *t*-test was used to compare the differences between the triplicate wells. A *p*-value less than 0.05 is considered to be significant in this study.

## Figures and Tables

**Table 1:** tDR expression in SARS-CoV-2-infected Calu-3 cells compared to mock infected controls.

Treatment Condition	Control Condition	tRF ID	Log2 Fold Change	Adjusted *p*-Value
SARS-CoV-2 24 h	mock 4 h	5028c	2.97	0.001
SARS-CoV-2 24 h	mock 4 h	5027c	−4.89	<0.001
SARS-CoV-2 24 h	mock 4 h	5020a	2.34	0.005
SARS-CoV-2 24 h	mock 4 h	5008c	−1.60	0.025

## Data Availability

Publicly archived datasets analyzed in this study are available at NCBI (Bioprojects PRJNA702830 and PRJNA702830). In addition, the tRNA fragments were obtained from http://genome.bioch.virginia.edu/trfdb/ accessed 17 January 2022.

## Supplementary Materials

The following supporting information can be downloaded at: https://www.mdpi.com/article/10.3390/pathogens11121479/s1, File S1: Significantly altered tRFs (*p* < 0.05) in individuals older than 60 diagnosed with COVID-19 compared to individuals younger than 60 diagnosed with COVID-19; File S2: Significantly altered tRFs (*p* < 0.05) in individuals less than 60 diagnosed with COVID-19 compared to uninfected individuals less than 60 years old; File S3: Significantly altered tRFs (*p* < 0.05) by gender; File S4: tDR-derived fragments’ homology in tDR-Namer; File S5: tDR-predicted binding to SARS-CoV-2 and VOCs.
