# Supplementary material for: Host tRNA-Derived RNAs Target the 3′Untranslated Region of SARS-CoV-2
Source: Pathogens. 2022 Dec 6;11(12):1479. doi: 10.3390/pathogens11121479 (PMC9786188; doi:10.3390/pathogens11121479)
Supplement: Supplementary file 1 [file pathogens-11-01479-s001.zip › Supplemental 4.pdf]

Homology to human tRNAs using tDRnamer
http://trna.ucsc.edu/tDRnamer/index.html

tDR-Gly GCGTTGGTGGTATAGTGGTGAGCATAGCTG

---

tDR-1:30-Gly-TCC-2 (tRF-Gly)

Organism	Homo sapiens (hg38 - GRCh38 Dec 2013)
tDR name	tDR-1:30-Gly-TCC-2
tDR sequence	..........|..........|..........| 5' GCGUUGGUGGUAUAGUGGUGAGCAUAGCUG
tDR sequence length	30
Sprinzl Position	1 to 30 (Acceptor stem to Anticodon stem)
Source tRNA	tRNA-Gly-TCC-2
Source type	mature tRNA
Isotype	Gly
Anticodon	TCC
# of mapped isodecoders	1
# of mismatches	0
# of indels	0

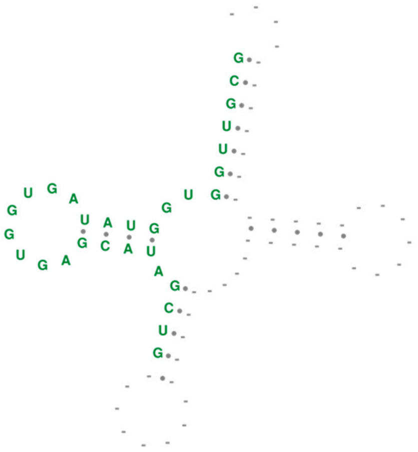

The alignment of the tDR-Gly to tRNA-Gly.

tDR-Val GTTTCCGTAGTGTAGTGGTTATCACGTTCGC

tDR-1:31-Val-AAC-1-M6 (tRF-Val)

Organism	Homo sapiens (hg38 - GRCh38 Dec 2013)
tDR name	tDR-1:31-Val-AAC-1-M6
Synonyms	tDR-1:31-Val-AAC-3-M6<br>tDR-1:31-Val-AAC-4-M6<br>tDR-1:31-Val-CAC-1-M6<br>tDR-1:31-Val-CAC-4-M6<br>tDR-1:31-Val-CAC-5-M6
tDR sequence	5' GUUUCCGUAGUGUAGUGGUUAUCACGUUCGC
tDR sequence length	31
Sprinzl Position	1 to 31 (Acceptor stem to Anticodon stem)
Source tRNAs	tRNA-Val-AAC-1<br>tRNA-Val-AAC-3<br>tRNA-Val-AAC-4<br>tRNA-Val-CAC-1<br>tRNA-Val-CAC-4<br>tRNA-Val-CAC-5
Source type	mature tRNA
Isotype	Val
Anticodon	CAC, AAC
# of mapped isodecoders	6
# of mismatches	0
# of indels	0

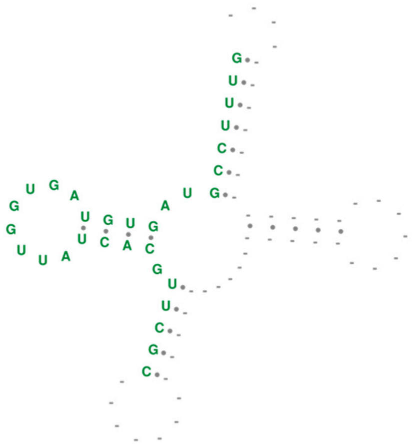

The alignment of the tDR-Val to tRNA-Val.
