# Supplementary material for: Host tRNA-Derived RNAs Target the 3′Untranslated Region of SARS-CoV-2
Source: Pathogens. 2022 Dec 6;11(12):1479. doi: 10.3390/pathogens11121479 (PMC9786188; doi:10.3390/pathogens11121479)
Supplement: Supplementary file 1 [file pathogens-11-01479-s001.zip › Supplemental 5.pdf]

RNAhybrid checked on 6-28-22
https://bibiserv.cebitec.uni-bielefeld.de/rnahybrid?viewType=submission&subType=rnahybrid_function_rnahybrid_p_1

for tDR-Gly (5'- GCGTTGGTGGTATAGTGGTGAGCATAGCTG)

for tDR-Val (5'-GTTTCCGTAGTGTAGTGGTTATCACGTTCGC

The reference sequence Reference (NC_045512.2)

Index Sequence top 25 tDR-Val

```
Version: RNAhybrid 2.2
Command line:/vol/bioapps/bin/RNAhybrid.bin -t
/var/bibiserv2/anonymous/rnahybrid/28/15/08/bibiserv2_2022-06-
28_150830_318kK/rnahybrid_input_target_rna_sequences_.file -s 3utr_human -q
/var/bibiserv2/anonymous/rnahybrid/28/15/08/bibiserv2_2022-06-
28_150830_318kK/rnahybrid_input_mirna_sequences.file -b 25 -n 31 -m 29903
searching
dataset: 1
mde of 5027: -64.200005
Individual hits
------------------------------------------------------------

dataset: 1
target: NC_045512.2
length: 29903
miRNA : 5027
length: 31

mfe: -31.0 kcal/mol
p-value: 1.000000e+00

position  6158
target 5' G                  GAUUAUAA       A      3'
           GUG AUGUGGUGGCUAUU        ACACUAC
           CGC UGCACUAUUGGUGA        UGUGAUG
miRNA  3'     U                             CCUUUG 5'

------------------------------------------------------------

dataset: 1
target: NC_045512.2
length: 29903
miRNA : 5027
length: 31

mfe: -30.9 kcal/mol
p-value: 1.000000e+00

position  29704
target 5'  A    U   AAG      C     UUCACCGAGGCCAC      U  G 3'
            GGAC UGA   AGCCAC ACAUU              GCGGAG AC
```

```
            CUUG ACU   UUGGUG UGUGA              UGCCUU UG
miRNA  3' CG    C   A        A                              5'

------------------------------------------------------------

dataset: 1
target: NC_045512.2
length: 29903
miRNA : 5027
length: 31

mfe: -30.5 kcal/mol
p-value: 1.000000e+00

position  5507
target 5' U           UGUAAA   UG  G  A  A C    A 3'
           UGAACGUGGUG      ACU  UG AC AC G AGAC
           GCUUGCACUAU      UGG  AU UG UG C UUUG
miRNA  3' C                    UG  G  A  C        5'

------------------------------------------------------------

dataset: 1
target: NC_045512.2
length: 29903
miRNA : 5027
length: 31

mfe: -30.0 kcal/mol
p-value: 1.000000e+00

position  6453
target 5' A   U       A      CGAAG UG         A 3'
           GUG AAUGUGA AACUAC     U  UA GGAGAC
           CGC UUGCACU UUGGUG     G  AU CCUUUG
miRNA  3'             A      AU    UG  G        5'

------------------------------------------------------------

dataset: 1
target: NC_045512.2
length: 29903
miRNA : 5027
length: 31

mfe: -29.9 kcal/mol
p-value: 1.000000e+00

position  29253
target 5' C G        U            A    U   C 3'
           G GAACGUGG UGACC  UACAC   GG  GC
           C CUUGCACU AUUGG  AUGUG   CC  UG
miRNA  3'   G              UG     AUG  UU    5'

------------------------------------------------------------

dataset: 1
```

```
target: NC_045512.2
length: 29903
miRNA : 5027
length: 31

mfe: -29.6 kcal/mol
p-value: 1.000000e+00

position  12237
target 5' U   C       CA     GCA  GUAAGU      A 3'
           UGA CGUGAUG  GCCAU   AC      UGGAAA
           GCU GCACUAU  UGGUG   UG      GCCUUU
miRNA  3' C   U              AUG  AU          G 5'

------------------------------------------------------------

dataset: 1
target: NC_045512.2
length: 29903
miRNA : 5027
length: 31

mfe: -29.3 kcal/mol
p-value: 1.000000e+00

position  10117
target 5'     U       CA         UA    UCUUU   U 3'
               UGUGGUA   ACUACACU  ACGG     GGC
               GCACUAU   UGAUGUGA  UGCC     UUG
miRNA  3' CGCUU       UGG              U         5'

------------------------------------------------------------

dataset: 1
target: NC_045512.2
length: 29903
miRNA : 5027
length: 31

mfe: -29.2 kcal/mol
p-value: 1.000000e+00

position  10668
target 5'   U   U    UGUA      UG UAUAA        A 3'
             AGC UGGU    CGCUGC  U     AUGGAGAC
             UUG ACUA    GUGAUG  A     UGCCUUUG
miRNA  3' CGC   C    UUG       UG                5'

------------------------------------------------------------

dataset: 1
target: NC_045512.2
length: 29903
miRNA : 5027
length: 31

mfe: -29.0 kcal/mol
```

```
p-value: 1.000000e+00

position  1457
target 5' C  A  G     CGC           UU      U 3'
           GU AG GUGGU   ACUAUUGC CU  GGAGGC
           CG UU CACUA   UGGUGAUG GA  CCUUUG
miRNA  3'    C  G     U          U  UG        5'

------------------------------------------------------------

dataset: 1
target: NC_045512.2
length: 29903
miRNA : 5027
length: 31

mfe: -29.0 kcal/mol
p-value: 1.000000e+00

position  8780
target 5' A  C        GG AG        AU  CAA   U 3'
           GC AGCGUGGU  U  UUAUACUA  GA   AGC
           CG UUGCACUA  G  GAUGUGAU  CU   UUG
miRNA  3'    C        UU GU        GC          5'

------------------------------------------------------------

dataset: 1
target: NC_045512.2
length: 29903
miRNA : 5027
length: 31

mfe: -29.0 kcal/mol
p-value: 1.000000e+00

position  29096
target 5' G  A            GA   AACCCAA      U 3'
           GC GACGUGGU  CCA  ACA       GGAAAU
           CG UUGCACUA  GGU  UGU       CCUUUG
miRNA  3'    C        UU   GA   GAUG          5'

------------------------------------------------------------

dataset: 1
target: NC_045512.2
length: 29903
miRNA : 5027
length: 31

mfe: -28.7 kcal/mol
p-value: 1.000000e+00

position  6366
target 5' A G                        A    C 3'
           G GAAUG GAUAAUC UUGC CUGCG AGAU
           C CUUGC CUAUUGG GAUG GAUGC UUUG
```

```
miRNA  3'   G     A       U    U     C      5'

------------------------------------------------------------

dataset: 1
target: NC_045512.2
length: 29903
miRNA : 5027
length: 31

mfe: -28.6 kcal/mol
p-value: 1.000000e+00

position  4158
target 5'     C      UAU   U   AAAAAGGCUGGUG       U     G 3'
               UGUGGU   ACC ACU             GCACUAC GAAAU
               GCACUA   UGG UGA             UGUGAUG CUUUG
miRNA  3' CGCUU      U                             C       5'

------------------------------------------------------------

dataset: 1
target: NC_045512.2
length: 29903
miRNA : 5027
length: 31

mfe: -28.4 kcal/mol
p-value: 1.000000e+00

position  27073
target 5' C         UAGCAG      CAGGUUUU     AUACAGUC     A   UU   A 3'
           GC AGCGUG      GUGACU        GCUGC        GCUAC GGA  GGC
           CG UUGCAC      UAUUGG        UGAUG        UGAUG CCU  UUG
miRNA  3'    C                                                       5'

------------------------------------------------------------

dataset: 1
target: NC_045512.2
length: 29903
miRNA : 5027
length: 31

mfe: -28.0 kcal/mol
p-value: 1.000000e+00

position  2246
target 5' G      AAAU  UC    UG    AA        U 3'
           GUGGAC    UG   ACC  UGCA    GGAAAU
           CGCUUG    AC   UGG  AUGU    CCUUUG
miRNA  3'        C     UAU   UG    GAUG        5'

------------------------------------------------------------

dataset: 1
target: NC_045512.2
```

```
length: 29903
miRNA : 5027
length: 31

mfe: -27.8 kcal/mol
p-value: 1.000000e+00

position  8700
target 5' U   UG            CGUGACAUA   U    A  U  U 3'
           UGA  GUGGUG UCACU         GCA CUAC GA AC
           GCU  CACUAU GGUGA         UGU GAUG CU UG
miRNA  3' C   UG      U                      C  U    5'

------------------------------------------------------------

dataset: 1
target: NC_045512.2
length: 29903
miRNA : 5027
length: 31

mfe: -27.7 kcal/mol
p-value: 1.000000e+00

position  653
target 5'  G              A         C    3'
            GAGC UGGUGGCCAU   GUUACGG
            CUUG ACUAUUGGUG   UGAUGCC
miRNA  3' CG    C          AUG       UUUG 5'

------------------------------------------------------------

dataset: 1
target: NC_045512.2
length: 29903
miRNA : 5027
length: 31

mfe: -27.7 kcal/mol
p-value: 1.000000e+00

position  28592
target 5'  A  UG     UU               C  3'
            GA  GU AU  CUACUAC CUA GGAA
            CU  CA UA  GGUGAUG GAU CCUU
miRNA  3' CG  UG  C  UU       U   G    UG 5'

------------------------------------------------------------

dataset: 1
target: NC_045512.2
length: 29903
miRNA : 5027
length: 31

mfe: -27.7 kcal/mol
p-value: 1.000000e+00
```

```
position  17157
target 5' A  UU  UCUCAU      GUUGAU        U    A 3'
           GC  GC      GCCGCU      GCACUAUG GAGA
           CG  UG      UGGUGA      UGUGAUGC CUUU
miRNA  3'    CU  CACUAU                         G 5'
```

```
dataset: 1
target: NC_045512.2
length: 29903
miRNA : 5027
length: 31

mfe: -27.7 kcal/mol
p-value: 1.000000e+00

position  19843
target 5' U  GG     ACAUU      A      UGAUC     CU  A 3'
           UG  UGUGG     GCUGCU AUACUG     UGGGA  AC
           GC  GCACU     UGGUGA UGUGAU     GCCUU  UG
miRNA  3' C  UU     AU                                5'
```

```
dataset: 1
target: NC_045512.2
length: 29903
miRNA : 5027
length: 31

mfe: -27.7 kcal/mol
p-value: 1.000000e+00

position  776
target 5' C    ACUCAU      GCU    GGAGGGGCAUA            UC   A 3'
           GUGA      GCGUGA   UAAC           CACU CGCUAUG  GAU
           CGCU      UGCACU   AUUG           GUGA GUGAUGC  UUG
miRNA  3'                                        U       CU     5'
```

```
dataset: 1
target: NC_045512.2
length: 29903
miRNA : 5027
length: 31

mfe: -27.6 kcal/mol
p-value: 1.000000e+00

position  25931
target 5' C     A   CU    GAUUGGUGG       GAAAA        C 3'
           UGAAC UGA  ACCA         UUAUACU     AUGGGAAU
           GCUUG ACU  UGGU         GAUGUGA     UGCCUUUG
miRNA  3' C     C   AU                                   5'
```

```
------------------------------------------------------------

dataset: 1
target: NC_045512.2
length: 29903
miRNA : 5027
length: 31

mfe: -27.4 kcal/mol
p-value: 1.000000e+00

position  3519
target 5' U     C          AUA     UAAU   C   3'
           UGAAU UGAUGAUUAC   GCUAC    GGA
           GCUUG ACUAUUGGUG   UGAUG    CCU
miRNA  3' C     C          AUG            UUG 5'

------------------------------------------------------------

dataset: 1
target: NC_045512.2
length: 29903
miRNA : 5027
length: 31

mfe: -27.4 kcal/mol
p-value: 1.000000e+00

position  4590
target 5' A    ACUCU   UACAAU      UG      U    A 3'
           UGAA     UGU      GCCACU  GCUAUG  AAC
           GCUU     GCA      UGGUGA  UGAUGC  UUG
miRNA  3' C            CUAU        UG      CU     5'

------------------------------------------------------------

dataset: 1
target: NC_045512.2
length: 29903
miRNA : 5027
length: 31

mfe: -27.3 kcal/mol
p-value: 1.000000e+00

position  335
target 5' C         CUCG    GUG   UU      U 3'
           GCG ACGUG     UAC   GCU  GGAGAC
           CGC UGCAC     GUG   UGA  CCUUUG
miRNA  3'    U     UAUUG   AUG   UG        5'

------------------------------------------------------------
```

Index sequence top 25 tDR-Gly

```
Version: RNAhybrid 2.2
Command line:/vol/bioapps/bin/RNAhybrid.bin -m 29903 -n 30 -t
/var/bibiserv2/anonymous/rnahybrid/28/15/46/bibiserv2_2022-06-
28_154601_KSR7p/rnahybrid_input_target_rna_sequences_.file -b 25 -q
/var/bibiserv2/anonymous/rnahybrid/28/15/46/bibiserv2_2022-06-
28_154601_KSR7p/rnahybrid_input_mirna_sequences.file -s 3utr_human
searching
dataset: 1
mde of 5008c: -65.000008
Individual hits
------------------------------------------------------------

dataset: 1
target: NC_045512.2
length: 29903
miRNA : 5008c
length: 30

mfe: -35.7 kcal/mol
p-value: 1.000000e+00

position  1486
target 5' A        GUUCUCUUAUGUUGG       AACAAG      UAUUGGGUUCCACGU        U
3'
           GGCUGUGU               UUGCCAU      UGUGCC               GCUAGCGC
           UCGAUACG               AGUGGUG      AUAUGG               UGGUUGCG
miRNA  3' G
5'

------------------------------------------------------------

dataset: 1
target: NC_045512.2
length: 29903
miRNA : 5008c
length: 30

mfe: -35.3 kcal/mol
p-value: 1.000000e+00

position  10397
target 5' U     UGUUACAAUG        U   GUGU          G 3'
           UAGCU          GUUCACCA CUG    UUACCAAUGU
           GUCGA          CGAGUGGU GAU    GGUGGUUGCG
miRNA  3'      UA                    AU              5'

------------------------------------------------------------

dataset: 1
target: NC_045512.2
length: 29903
miRNA : 5008c
length: 30
```

```
mfe: -35.2 kcal/mol
p-value: 1.000000e+00

position  17155
target 5' A                         GUU     A 3'
           CAGCU UGCUC UCAU   GCCGCU   GAUGC
           GUCGA ACGAG GGUG   UGGUGG   UUGCG
miRNA  3'       U     U   AUA                 5'

------------------------------------------------------------

dataset: 1
target: NC_045512.2
length: 29903
miRNA : 5008c
length: 30

mfe: -33.0 kcal/mol
p-value: 1.000000e+00

position  14574
target 5'  U       A       UCUGGUAAUC        GAUAA    A 3'
            GCUAUGC CGCUGCU          UAUUACUA     ACGC
            CGAUACG GUGGUGA          AUGGUGGU     UGCG
miRNA  3' GU       A       U                            5'

------------------------------------------------------------

dataset: 1
target: NC_045512.2
length: 29903
miRNA : 5008c
length: 30

mfe: -32.9 kcal/mol
p-value: 1.000000e+00

position  13760
target 5' A    GAC   G       A        GU      C 3'
           CGGU   AUG  UACCAC UAUAUCAC  CAACGU
           GUCG   UAC  GUGGUG AUAUGGUG  GUUGCG
miRNA  3'      A     GA                         5'

------------------------------------------------------------

dataset: 1
target: NC_045512.2
length: 29903
miRNA : 5008c
length: 30

mfe: -32.7 kcal/mol
p-value: 1.000000e+00

position  17433
target 5' U   GACCC      AU        UG  CC    A 3'
           GGC     UGCUCA      UACC  CA  ACGC
```

```
           UCG     ACGAGU      AUGG  GU  UGCG
miRNA  3' G   AU         GGUGAU    UG          5'

------------------------------------------------------------

dataset: 1
target: NC_045512.2
length: 29903
miRNA : 5008c
length: 30

mfe: -32.5 kcal/mol
p-value: 1.000000e+00

position  28420
target 5'  U  GUCUUG          CUC  U  A  UG  A 3'
            GC      GUUCACCGCU   AC CA CA  GC
            CG      CGAGUGGUGA   UG GU GU  CG
miRNA  3' GU  AUA             UA      G  UG    5'

------------------------------------------------------------

dataset: 1
target: NC_045512.2
length: 29903
miRNA : 5008c
length: 30

mfe: -32.4 kcal/mol
p-value: 1.000000e+00

position  29506
target 5' G   A     G   CAACUCAGGCCUAA   CAUGCAG     A   G  A 3'
           AGC GUGCU ACU              ACU       ACCAC CAA GC
           UCG UACGA UGG              UGA       UGGUG GUU CG
miRNA  3' G   A     G                    UA              G    5'

------------------------------------------------------------

dataset: 1
target: NC_045512.2
length: 29903
miRNA : 5008c
length: 30

mfe: -32.3 kcal/mol
p-value: 1.000000e+00

position  26706
target 5' U     UGUUUU              UU  AGAAUAAAUUGGAU    GG  G 3'
           UAGCU      GUGCUUGCUGCUGU  AC              CACC  UG
           GUCGA      UACGAGUGGUGAUA  UG              GUGG  GC
miRNA  3'                                                 UU  G 5'

------------------------------------------------------------

dataset: 1
```

```
target: NC_045512.2
length: 29903
miRNA : 5008c
length: 30

mfe: -32.3 kcal/mol
p-value: 1.000000e+00

position  24226
target 5' U   GCAG     G      AA        UGCU    A 3'
           GGU    GUGCU CAUUAC  AUACCAUU    AUGC
           UCG    UACGA GUGGUG  UAUGGUGG    UGCG
miRNA  3' G   A               A         U         5'

------------------------------------------------------------

dataset: 1
target: NC_045512.2
length: 29903
miRNA : 5008c
length: 30

mfe: -32.0 kcal/mol
p-value: 1.000000e+00

position  4679
target 5' C      CAGUUUC   UUCU     UG      GUUA      A 3'
           CAGCUA       UGU    UCACC   AUGCU    CAGCGU
           GUCGAU       ACG    AGUGG   UAUGG    GUUGCG
miRNA  3'                           UGA     UG          5'

------------------------------------------------------------

dataset: 1
target: NC_045512.2
length: 29903
miRNA : 5008c
length: 30

mfe: -32.0 kcal/mol
p-value: 1.000000e+00

position  26800
target 5' U      CUUCAU        UUCAG    U UG   GU    G 3'
           CAGCUA      UGCUU CU      ACUG U  CGC  ACGC
           GUCGAU      ACGAG GG      UGAU A  GUG  UGCG
miRNA  3'                   U             UG   GU      5'

------------------------------------------------------------

dataset: 1
target: NC_045512.2
length: 29903
miRNA : 5008c
length: 30

mfe: -31.7 kcal/mol
```

p-value: 1.000000e+00

position 16927
```
target 5' A    A    CAUUAAGUG    UAC    G      AAGA  A 3'
           CAGU AUGC         CACC   ACUA UGCCAC    GC
           GUCG UACG         GUGG   UGAU AUGGUG    CG
miRNA  3'      A    A                          GUUG    5'
```

----

dataset: 1
target: NC_045512.2
length: 29903
miRNA : 5008c
length: 30

mfe: -31.7 kcal/mol
p-value: 1.000000e+00

position 4179
```
target 5' A        GG        AAA        A 3'
           GGCUG GU  CACUACUG   UGCUAGCG
           UCGAU CG  GUGGUGAU   GUGGUUGC
miRNA  3' G     A  A         AUG        G 5'
```

----

dataset: 1
target: NC_045512.2
length: 29903
miRNA : 5008c
length: 30

mfe: -31.6 kcal/mol
p-value: 1.000000e+00

position 1317
```
target 5' A  AAG            UGUGGUUACU        AA    U 3'
           AG   GUGC CACUACU          UACC CCA  AUGC
           UC   UACG GUGGUGA          AUGG GGU  UGCG
miRNA  3' G  GA     A       U             U           5'
```

----

dataset: 1
target: NC_045512.2
length: 29903
miRNA : 5008c
length: 30

mfe: -31.6 kcal/mol
p-value: 1.000000e+00

position 29174
```
target 5' U   C    AAA UG   AAUU    C       U 3'
           GGC   GC   U  CAC    UGCC CCAGCGC
           UCG   CG   G  GUG    AUGG GGUUGCG
```

```
miRNA  3' G   AUA  A   UG   AU      U         5'

------------------------------------------------------------

dataset: 1
target: NC_045512.2
length: 29903
miRNA : 5008c
length: 30

mfe: -31.5 kcal/mol
p-value: 1.000000e+00

position  26994
target 5'  C    UGACAUCAAGGACC   CUAAAGAAA     GUU    C     G     U 3'
            GCUG              UGC         UCACU   GCUA AUCAC AACGC
            CGAU              ACG         AGUGG   UGAU UGGUG UUGCG
miRNA  3' GU                                          A     G       5'

------------------------------------------------------------

dataset: 1
target: NC_045512.2
length: 29903
miRNA : 5008c
length: 30

mfe: -31.5 kcal/mol
p-value: 1.000000e+00

position  14789
target 5' U    G  UA G        CGUUAUAAU        A  3'
           CAGC AU  U ACUACUAU         CUACCAAC
           GUCG UA  A UGGUGAUA         GGUGGUUG
miRNA  3'      A  CG G        U                CG 5'

------------------------------------------------------------

dataset: 1
target: NC_045512.2
length: 29903
miRNA : 5008c
length: 30

mfe: -31.4 kcal/mol
p-value: 1.000000e+00

position  16205
target 5' A        ACA           AGU  UAC G  U 3'
           GGCUAUGU   CACCGC AUAC   CU   A GC
           UCGAUACG   GUGGUG UAUG   GG   U CG
miRNA  3' G        A        A    GU   U   G    5'

------------------------------------------------------------

dataset: 1
target: NC_045512.2
```

```
length: 29903
miRNA : 5008c
length: 30

mfe: -31.4 kcal/mol
p-value: 1.000000e+00

position  29716
target 5'       A         AUUU       G  C 3'
                 GC CACCAC    UCACCGA GC
                 CG GUGGUG    GGUGGUU CG
miRNA  3' GUCGAUA  A      AUAU       G    5'

------------------------------------------------------------

dataset: 1
target: NC_045512.2
length: 29903
miRNA : 5008c
length: 30

mfe: -31.3 kcal/mol
p-value: 1.000000e+00

position  11107
target 5'  U      GGUA           U     UU   A 3'
            GCUAUG    UUAUUGCUAUG CUGCU  UGC
            CGAUAC    AGUGGUGAUAU GGUGG  GCG
miRNA  3' GU      G                    UU     5'

------------------------------------------------------------

dataset: 1
target: NC_045512.2
length: 29903
miRNA : 5008c
length: 30

mfe: -31.2 kcal/mol
p-value: 1.000000e+00

position  13228
target 5' U   G    A   UGU GU           U   C 3'
           GGU GUGC UCG   U  CUGUACUGCCG UGC
           UCG UACG AGU   G  GAUAUGGUGGU GCG
miRNA  3' G   A            GU           U     5'

------------------------------------------------------------

dataset: 1
target: NC_045512.2
length: 29903
miRNA : 5008c
length: 30

mfe: -31.1 kcal/mol
p-value: 1.000000e+00
```

```
position  1178
target 5' C         G      AAUGA    A     AUG   C 3'
           CAGU  UGC UCACCA     AUGC ACCAA   UGC
           GUCG  ACG AGUGGU     UAUG UGGUU   GCG
miRNA  3'      AU          GA       G             5'
```

```
dataset: 1
target: NC_045512.2
length: 29903
miRNA : 5008c
length: 30

mfe: -31.1 kcal/mol
p-value: 1.000000e+00

position  2865
target 5' A   AAA  A       UG   UG  G  G    U 3'
           AGU   UG GUUCGCC  UGU  UG CA AUGC
           UCG   AU CGAGUGG  AUA  GU GU UGCG
miRNA  3' G        A       UG   UG  G         5'
```

```
dataset: 1
target: NC_045512.2
length: 29903
miRNA : 5008c
length: 30

mfe: -30.9 kcal/mol
p-value: 1.000000e+00

position  18233
target 5' A    A   G         ACAUGUU       C   A 3'
           AGUU AUG UUACC CUA       UAUCACC GCG
           UCGA UAC AGUGG GAU       AUGGUGG UGC
miRNA  3' G        G     U                 U   G 5'
```

Alpha tDR-Val

Version: RNAhybrid 2.2

```
Command line:/vol/bioapps/bin/RNAhybrid.bin -s 3utr_human -t
/var/bibiserv2/anonymous/rnahybrid/28/15/15/bibiserv2_2022-06-
28_151531_1DZlc/rnahybrid_input_target_rna_sequences_.file -m 29851 -q
/var/bibiserv2/anonymous/rnahybrid/28/15/15/bibiserv2_2022-06-
28_151531_1DZlc/rnahybrid_input_mirna_sequences.file -b 25 -n 31
searching
dataset: 1
mde of 5027: -64.200005
Individual hits
------------------------------------------------------------

dataset: 1
target: OV054768.1
length: 29851
miRNA : 5027
length: 31

mfe: -31.0 kcal/mol
p-value: 1.000000e+00

position  6158
target 5' G                  GAUUAUAA       A      3'
           GUG AUGUGGUGGCUAUU        ACACUAC
           CGC UGCACUAUUGGUGA        UGUGAUG
miRNA  3'     U                             CCUUUG 5'

------------------------------------------------------------

dataset: 1
target: OV054768.1
length: 29851
miRNA : 5027
length: 31

mfe: -30.9 kcal/mol
p-value: 1.000000e+00

position  29685
target 5'  A    U   AAG      C     UUCACCGAGGCCAC      U  G 3'
            GGAC UGA   AGCCAC ACAUU              GCGGAG AC
            CUUG ACU   UUGGUG UGUGA              UGCCUU UG
miRNA  3' CG    C   A        A                              5'

------------------------------------------------------------

dataset: 1
target: OV054768.1
length: 29851
miRNA : 5027
length: 31

mfe: -30.5 kcal/mol
p-value: 1.000000e+00

position  5507
target 5' U           UGUAAA   UG  G  A  A C    A 3'
           UGAACGUGGUG      ACU  UG AC AC G AGAC
```

```
           GCUUGCACUAU      UGG  AU UG UG C UUUG
miRNA  3' C                    UG  G  A  C        5'
```

----

dataset: 1
target: OV054768.1
length: 29851
miRNA : 5027
length: 31

mfe: -30.0 kcal/mol
p-value: 1.000000e+00

position 6453
```
target 5' A   U       A      CGAAG UG         A 3'
           GUG AAUGUGA AACUAC     U  UA GGAGAC
           CGC UUGCACU UUGGUG     G  AU CCUUUG
miRNA  3'             A      AU    UG  G        5'
```

----

dataset: 1
target: OV054768.1
length: 29851
miRNA : 5027
length: 31

mfe: -29.9 kcal/mol
p-value: 1.000000e+00

position 29234
```
target 5' C G        U            A    U   C 3'
           G GAACGUGG UGACC  UACAC   GG  GC
           C CUUGCACU AUUGG  AUGUG   CC  UG
miRNA  3'   G              UG     AUG  UU    5'
```

----

dataset: 1
target: OV054768.1
length: 29851
miRNA : 5027
length: 31

mfe: -29.6 kcal/mol
p-value: 1.000000e+00

position 12228
```
target 5' U   C       CA     GCA  GUAAGU      A 3'
           UGA CGUGAUG  GCCAU   AC      UGGAAA
           GCU GCACUAU  UGGUG   UG      GCCUUU
miRNA  3' C   U              AUG  AU          G 5'
```

----

dataset: 1

target: OV054768.1
length: 29851
miRNA : 5027
length: 31

mfe: -29.3 kcal/mol
p-value: 1.000000e+00

position 10117
```
target 5'     U       CA         UA    UCUUU   U 3'
               UGUGGUA   ACUACACU  ACGG     GGC
               GCACUAU   UGAUGUGA  UGCC     UUG
miRNA  3' CGCUU       UGG             U         5'
```

----

dataset: 1
target: OV054768.1
length: 29851
miRNA : 5027
length: 31

mfe: -29.2 kcal/mol
p-value: 1.000000e+00

position 10668
```
target 5'   U   U    UGUA      UG UAUAA        A 3'
             AGC UGGU    CGCUGC  U     AUGGAGAC
             UUG ACUA    GUGAUG  A     UGCCUUUG
miRNA  3' CGC   C    UUG       UG                5'
```

----

dataset: 1
target: OV054768.1
length: 29851
miRNA : 5027
length: 31

mfe: -29.0 kcal/mol
p-value: 1.000000e+00

position 1457
```
target 5' C  A  G     CGC          UU      U 3'
           GU AG GUGGU   ACUAUUGC CU  GGAGGC
           CG UU CACUA   UGGUGAUG GA  CCUUUG
miRNA  3'    C  G     U          U  UG        5'
```

----

dataset: 1
target: OV054768.1
length: 29851
miRNA : 5027
length: 31

mfe: -29.0 kcal/mol

```
p-value: 1.000000e+00

position  8780
target 5' A  C        GG AG        AU  CAA   U 3'
           GC AGCGUGGU  U  UUAUACUA  GA   AGC
           CG UUGCACUA  G  GAUGUGAU  CU   UUG
miRNA  3'    C        UU GU        GC          5'

------------------------------------------------------------

dataset: 1
target: OV054768.1
length: 29851
miRNA : 5027
length: 31

mfe: -29.0 kcal/mol
p-value: 1.000000e+00

position  29077
target 5' G  A             GA   AACCCAA      U 3'
           GC GACGUGGU  CCA  ACA       GGAAAU
           CG UUGCACUA  GGU  UGU       CCUUUG
miRNA  3'    C        UU   GA   GAUG           5'

------------------------------------------------------------

dataset: 1
target: OV054768.1
length: 29851
miRNA : 5027
length: 31

mfe: -28.7 kcal/mol
p-value: 1.000000e+00

position  6366
target 5' A G                        A    C 3'
           G GAAUG GAUAAUC UUGC CUGCG AGAU
           C CUUGC CUAUUGG GAUG GAUGC UUUG
miRNA  3'   G     A       U    U     C      5'

------------------------------------------------------------

dataset: 1
target: OV054768.1
length: 29851
miRNA : 5027
length: 31

mfe: -28.6 kcal/mol
p-value: 1.000000e+00

position  4158
target 5'     C      UAU   U   AAAAAGGCUGGUG       U     G 3'
               UGUGGU   ACC ACU             GCACUAC GAAAU
               GCACUA   UGG UGA             UGUGAUG CUUUG
```

```
miRNA  3' CGCUU      U                             C       5'

------------------------------------------------------------

dataset: 1
target: OV054768.1
length: 29851
miRNA : 5027
length: 31

mfe: -28.4 kcal/mol
p-value: 1.000000e+00

position  27055
target 5' C         UAGCAG      CAGGUUUU     AUACAGUC     A   UU   A 3'
           GC AGCGUG      GUGACU        GCUGC        GCUAC GGA  GGC
           CG UUGCAC      UAUUGG        UGAUG        UGAUG CCU  UUG
miRNA  3'    C                                                       5'

------------------------------------------------------------

dataset: 1
target: OV054768.1
length: 29851
miRNA : 5027
length: 31

mfe: -28.0 kcal/mol
p-value: 1.000000e+00

position  2246
target 5' G      AAAU  UC    UG    AA        U 3'
           GUGGAC    UG   ACC  UGCA    GGAAAU
           CGCUUG    AC   UGG  AUGU    CCUUUG
miRNA  3'        C     UAU   UG    GAUG        5'

------------------------------------------------------------

dataset: 1
target: OV054768.1
length: 29851
miRNA : 5027
length: 31

mfe: -27.8 kcal/mol
p-value: 1.000000e+00

position  8700
target 5' U   UG            CGUGACAUA   U    A  U  U 3'
           UGA  GUGGUG UCACU         GCA CUAC GA AC
           GCU  CACUAU GGUGA         UGU GAUG CU UG
miRNA  3' C   UG      U                      C  U    5'

------------------------------------------------------------

dataset: 1
target: OV054768.1
```

length: 29851
miRNA : 5027
length: 31

mfe: -27.7 kcal/mol
p-value: 1.000000e+00

position 653
```
target 5'  G               A         C    3'
            GAGC UGGUGGCCAU   GUUACGG
            CUUG ACUAUUGGUG   UGAUGCC
miRNA  3' CG    C          AUG       UUUG 5'
```

----

dataset: 1
target: OV054768.1
length: 29851
miRNA : 5027
length: 31

mfe: -27.7 kcal/mol
p-value: 1.000000e+00

position 28573
```
target 5'  A  UG     UU                C  3'
            GA  GU AU  CUACUAC CUA GGAA
            CU  CA UA  GGUGAUG GAU CCUU
miRNA  3' CG  UG  C  UU       U   G    UG 5'
```

----

dataset: 1
target: OV054768.1
length: 29851
miRNA : 5027
length: 31

mfe: -27.7 kcal/mol
p-value: 1.000000e+00

position 17148
```
target 5' A  UU  UCUCAU      GUUGAU        U    A 3'
           GC  GC      GCCGCU      GCACUAUG GAGA
           CG  UG      UGGUGA      UGUGAUGC CUUU
miRNA  3'    CU  CACUAU                         G 5'
```

----

dataset: 1
target: OV054768.1
length: 29851
miRNA : 5027
length: 31

mfe: -27.7 kcal/mol
p-value: 1.000000e+00

```
position  19834
target 5' U  GG     ACAUU      A      UGAUC     CU  A 3'
           UG  UGUGG     GCUGCU AUACUG     UGGGA  AC
           GC  GCACU     UGGUGA UGUGAU     GCCUU  UG
miRNA  3' C  UU     AU                                5'

------------------------------------------------------------

dataset: 1
target: OV054768.1
length: 29851
miRNA : 5027
length: 31

mfe: -27.7 kcal/mol
p-value: 1.000000e+00

position  776
target 5' C    ACUCAU      GCU    GGAGGGGCAUA            UC   A 3'
           GUGA      GCGUGA   UAAC           CACU CGCUAUG  GAU
           CGCU      UGCACU   AUUG           GUGA GUGAUGC  UUG
miRNA  3'                                        U       CU     5'

------------------------------------------------------------

dataset: 1
target: OV054768.1
length: 29851
miRNA : 5027
length: 31

mfe: -27.6 kcal/mol
p-value: 1.000000e+00

position  25913
target 5' C     A   CU    GAUUGGUGG       GAAAA        C 3'
           UGAAC UGA  ACCA         UUAUACU     AUGGGAAU
           GCUUG ACU  UGGU         GAUGUGA     UGCCUUUG
miRNA  3' C     C   AU                                   5'

------------------------------------------------------------

dataset: 1
target: OV054768.1
length: 29851
miRNA : 5027
length: 31

mfe: -27.4 kcal/mol
p-value: 1.000000e+00

position  3519
target 5' U     C          AUA     UAAU   C   3'
           UGAAU UGAUGAUUAC   GCUAC    GGA
           GCUUG ACUAUUGGUG   UGAUG    CCU
miRNA  3' C     C          AUG            UUG 5'
```

```
------------------------------------------------------------

dataset: 1
target: OV054768.1
length: 29851
miRNA : 5027
length: 31

mfe: -27.4 kcal/mol
p-value: 1.000000e+00

position  4590
target 5' A    ACUCU   UACAAU      UG      U    A 3'
           UGAA     UGU      GCCACU  GCUAUG  AAC
           GCUU     GCA      UGGUGA  UGAUGC  UUG
miRNA  3' C            CUAU        UG      CU     5'

------------------------------------------------------------

dataset: 1
target: OV054768.1
length: 29851
miRNA : 5027
length: 31

mfe: -27.3 kcal/mol
p-value: 1.000000e+00

position  335
target 5' C         CUCG    GUG   UU      U 3'
           GCG ACGUG     UAC   GCU  GGAGAC
           CGC UGCAC     GUG   UGA  CCUUUG
miRNA  3'     U     UAUUG   AUG   UG        5'
```

Alpha top 25 tDR-Gly

```
Version: RNAhybrid 2.2
Command line:/vol/bioapps/bin/RNAhybrid.bin -n 30 -s 3utr_human -b 25 -t
/var/bibiserv2/anonymous/rnahybrid/28/15/33/bibiserv2_2022-06-
28_153336_fkmK8/rnahybrid_input_target_rna_sequences_.file -q
/var/bibiserv2/anonymous/rnahybrid/28/15/33/bibiserv2_2022-06-
28_153336_fkmK8/rnahybrid_input_mirna_sequences.file -m 29851
searching
dataset: 1
mde of 5008c: -65.000008
Individual hits
------------------------------------------------------------

dataset: 1
target: OV054768.1
length: 29851
miRNA : 5008c
length: 30

mfe: -35.7 kcal/mol
```

```
p-value: 1.000000e+00

position  1486
target 5' A        GUUCUCUUAUGUUGG       AACAAG      UAUUGGGUUCCACGU        U
3'
           GGCUGUGU               UUGCCAU      UGUGCC               GCUAGCGC
           UCGAUACG               AGUGGUG      AUAUGG               UGGUUGCG
miRNA  3' G
5'

------------------------------------------------------------

dataset: 1
target: OV054768.1
length: 29851
miRNA : 5008c
length: 30

mfe: -35.3 kcal/mol
p-value: 1.000000e+00

position  10397
target 5' U     UGUUACAAUG        U   GUGU          G 3'
           UAGCU          GUUCACCA CUG    UUACCAAUGU
           GUCGA          CGAGUGGU GAU    GGUGGUUGCG
miRNA  3'      UA                   AU              5'

------------------------------------------------------------

dataset: 1
target: OV054768.1
length: 29851
miRNA : 5008c
length: 30

mfe: -35.2 kcal/mol
p-value: 1.000000e+00

position  17146
target 5' A                         GUU     A 3'
           CAGCU UGCUC UCAU   GCCGCU   GAUGC
           GUCGA ACGAG GGUG   UGGUGG   UUGCG
miRNA  3'      U     U    AUA               5'

------------------------------------------------------------

dataset: 1
target: OV054768.1
length: 29851
miRNA : 5008c
length: 30

mfe: -33.0 kcal/mol
p-value: 1.000000e+00

position  14565
target 5'  U       A       UCUGGUAAUC        GAUAA    A 3'
```

```
            GCUAUGC CGCUGCU          UAUUACUA     ACGC
            CGAUACG GUGGUGA          AUGGUGGU     UGCG
miRNA  3' GU       A       U                            5'

------------------------------------------------------------

dataset: 1
target: OV054768.1
length: 29851
miRNA : 5008c
length: 30

mfe: -32.9 kcal/mol
p-value: 1.000000e+00

position  13751
target 5' A   GAC    G       A        GU      C 3'
           CGGU   AUG  UACCAC UAUAUCAC  CAACGU
           GUCG   UAC  GUGGUG AUAUGGUG  GUUGCG
miRNA  3'      A     GA                         5'

------------------------------------------------------------

dataset: 1
target: OV054768.1
length: 29851
miRNA : 5008c
length: 30

mfe: -32.7 kcal/mol
p-value: 1.000000e+00

position  17424
target 5' U   GACCC      AU        UG  CC    A 3'
           GGC     UGCUCA      UACC  CA  ACGC
           UCG     ACGAGU      AUGG  GU  UGCG
miRNA  3' G   AU         GGUGAU    UG          5'

------------------------------------------------------------

dataset: 1
target: OV054768.1
length: 29851
miRNA : 5008c
length: 30

mfe: -32.5 kcal/mol
p-value: 1.000000e+00

position  28401
target 5'  U  GUCUUG          CUC  U  A  UG  A 3'
            GC      GUUCACCGCU   AC CA CA  GC
            CG      CGAGUGGUGA   UG GU GU  CG
miRNA  3' GU  AUA             UA      G  UG    5'

------------------------------------------------------------
```

```
dataset: 1
target: OV054768.1
length: 29851
miRNA : 5008c
length: 30

mfe: -32.4 kcal/mol
p-value: 1.000000e+00

position  29487
target 5' G   A     G   CAACUCAGGCCUAA   CAUGCAG     A   G  A 3'
           AGC GUGCU ACU              ACU       ACCAC CAA GC
           UCG UACGA UGG              UGA       UGGUG GUU CG
miRNA  3' G   A     G                    UA              G    5'

------------------------------------------------------------

dataset: 1
target: OV054768.1
length: 29851
miRNA : 5008c
length: 30

mfe: -32.3 kcal/mol
p-value: 1.000000e+00

position  26688
target 5' U     UGUUUU              UU  AGAAUAAAUUGGAU    GG  G 3'
           UAGCU      GUGCUUGCUGCUGU  AC              CACC  UG
           GUCGA      UACGAGUGGUGAUA  UG              GUGG  GC
miRNA  3'                                                 UU  G 5'

------------------------------------------------------------

dataset: 1
target: OV054768.1
length: 29851
miRNA : 5008c
length: 30

mfe: -32.3 kcal/mol
p-value: 1.000000e+00

position  24208
target 5' U   GCAG     G      AA        UGCU    A 3'
           GGU    GUGCU CAUUAC  AUACCAUU    AUGC
           UCG    UACGA GUGGUG  UAUGGUGG    UGCG
miRNA  3' G   A               A         U         5'

------------------------------------------------------------

dataset: 1
target: OV054768.1
length: 29851
miRNA : 5008c
length: 30
```

```
mfe: -32.0 kcal/mol
p-value: 1.000000e+00

position  4679
target 5' C      CAGUUUC   UUCU     UG      GUUA      A 3'
           CAGCUA       UGU    UCACC   AUGCU    CAGCGU
           GUCGAU       ACG    AGUGG   UAUGG    GUUGCG
miRNA  3'                           UGA     UG          5'

------------------------------------------------------------

dataset: 1
target: OV054768.1
length: 29851
miRNA : 5008c
length: 30

mfe: -32.0 kcal/mol
p-value: 1.000000e+00

position  26782
target 5' U      CUUCAU        UUCAG    U UG   GU    G 3'
           CAGCUA      UGCUU CU     ACUG U  CGC  ACGC
           GUCGAU      ACGAG GG     UGAU A  GUG  UGCG
miRNA  3'                   U             UG   GU      5'

------------------------------------------------------------

dataset: 1
target: OV054768.1
length: 29851
miRNA : 5008c
length: 30

mfe: -31.8 kcal/mol
p-value: 1.000000e+00

position  16143
target 5'  U             AUG   A    UC  G  A 3'
            GUUAUGCUUACUA   AUA CACC  AA GU
            CGAUACGAGUGGU   UAU GUGG  UU CG
miRNA  3' GU             GA    G        G    5'

------------------------------------------------------------

dataset: 1
target: OV054768.1
length: 29851
miRNA : 5008c
length: 30

mfe: -31.7 kcal/mol
p-value: 1.000000e+00

position  16918
target 5' A    A    CAUUAAGUG    UAC    G      AAGA  A 3'
           CAGU AUGC         CACC   ACUA UGCCAC    GC
```

```
           GUCG UACG         GUGG   UGAU AUGGUG    CG
miRNA  3'      A    A                         GUUG    5'

------------------------------------------------------------

dataset: 1
target: OV054768.1
length: 29851
miRNA : 5008c
length: 30

mfe: -31.7 kcal/mol
p-value: 1.000000e+00

position  4179
target 5' A        GG        AAA        A 3'
           GGCUG GU  CACUACUG   UGCUAGCG
           UCGAU CG  GUGGUGAU   GUGGUUGC
miRNA  3' G     A  A         AUG        G 5'

------------------------------------------------------------

dataset: 1
target: OV054768.1
length: 29851
miRNA : 5008c
length: 30

mfe: -31.6 kcal/mol
p-value: 1.000000e+00

position  1317
target 5' A  AAG            UGUGGUUACU        AA    U 3'
           AG   GUGC CACUACU          UACC CCA  AUGC
           UC   UACG GUGGUGA          AUGG GGU  UGCG
miRNA  3' G  GA     A       U             U           5'

------------------------------------------------------------

dataset: 1
target: OV054768.1
length: 29851
miRNA : 5008c
length: 30

mfe: -31.6 kcal/mol
p-value: 1.000000e+00

position  29155
target 5' U   C    AAA UG   AAUU    C       U 3'
           GGC   GC   U  CAC    UGCC CCAGCGC
           UCG   CG   G  GUG    AUGG GGUUGCG
miRNA  3' G   AUA  A   UG   AU      U         5'

------------------------------------------------------------

dataset: 1
```

```
target: OV054768.1
length: 29851
miRNA : 5008c
length: 30

mfe: -31.5 kcal/mol
p-value: 1.000000e+00

position  26976
target 5'  C    UGACAUCAAGGACC   CUAAAGAAA     GUU    C     G     U 3'
            GCUG              UGC         UCACU   GCUA AUCAC AACGC
            CGAU              ACG         AGUGG   UGAU UGGUG UUGCG
miRNA  3' GU                                          A     G       5'

------------------------------------------------------------

dataset: 1
target: OV054768.1
length: 29851
miRNA : 5008c
length: 30

mfe: -31.5 kcal/mol
p-value: 1.000000e+00

position  14780
target 5' U    G  UA G        CGUUAUAAU        A  3'
           CAGC AU  U ACUACUAU         CUACCAAC
           GUCG UA  A UGGUGAUA         GGUGGUUG
miRNA  3'      A  CG G        U                CG 5'

------------------------------------------------------------

dataset: 1
target: OV054768.1
length: 29851
miRNA : 5008c
length: 30

mfe: -31.4 kcal/mol
p-value: 1.000000e+00

position  16196
target 5' A        ACA           AGU  UAC G  U 3'
           GGCUAUGU   CACCGC AUAC   CU   A GC
           UCGAUACG   GUGGUG UAUG   GG   U CG
miRNA  3' G        A        A    GU   U   G    5'

------------------------------------------------------------

dataset: 1
target: OV054768.1
length: 29851
miRNA : 5008c
length: 30

mfe: -31.4 kcal/mol
```

```
p-value: 1.000000e+00

position  29697
target 5'       A         AUUU       G  C 3'
                 GC CACCAC    UCACCGA GC
                 CG GUGGUG    GGUGGUU CG
miRNA  3' GUCGAUA  A      AUAU       G    5'

------------------------------------------------------------

dataset: 1
target: OV054768.1
length: 29851
miRNA : 5008c
length: 30

mfe: -31.3 kcal/mol
p-value: 1.000000e+00

position  11107
target 5'  U      GGUA           U     UU   A 3'
            GCUAUG    UUAUUGCUAUG CUGCU  UGC
            CGAUAC    AGUGGUGAUAU GGUGG  GCG
miRNA  3' GU      G                    UU     5'

------------------------------------------------------------

dataset: 1
target: OV054768.1
length: 29851
miRNA : 5008c
length: 30

mfe: -31.2 kcal/mol
p-value: 1.000000e+00

position  13219
target 5' U   G    A   UGU GU           U   C 3'
           GGU GUGC UCG   U  CUGUACUGCCG UGC
           UCG UACG AGU   G  GAUAUGGUGGU GCG
miRNA  3' G   A            GU           U     5'

------------------------------------------------------------

dataset: 1
target: OV054768.1
length: 29851
miRNA : 5008c
length: 30

mfe: -31.1 kcal/mol
p-value: 1.000000e+00

position  1178
target 5' C         G      AAUGA    A     AUG   C 3'
           CAGU  UGC UCACCA     AUGC ACCAA   UGC
           GUCG  ACG AGUGGU     UAUG UGGUU   GCG
```

```
miRNA  3'      AU          GA       G             5'

------------------------------------------------------------

dataset: 1
target: OV054768.1
length: 29851
miRNA : 5008c
length: 30

mfe: -31.1 kcal/mol
p-value: 1.000000e+00

position  2865
target 5' A   AAA  A       UG   UG  G  G    U 3'
           AGU   UG GUUCGCC  UGU  UG CA AUGC
           UCG   AU CGAGUGG  AUA  GU GU UGCG
miRNA  3' G        A       UG   UG  G         5'

-----------------------------
```

Delta top 25 tDR-Val

```
Version: RNAhybrid 2.2
Command line:/vol/bioapps/bin/RNAhybrid.bin -t
/var/bibiserv2/anonymous/rnahybrid/28/15/00/bibiserv2_2022-06-
28_150030_iiI5m/rnahybrid_input_target_rna_sequences_.file -s 3utr_human -n
31 -b 25 -m 29836 -q
/var/bibiserv2/anonymous/rnahybrid/28/15/00/bibiserv2_2022-06-
28_150030_iiI5m/rnahybrid_input_mirna_sequences.file
searching
dataset: 1
mde of 5027: -64.200005
Individual hits
------------------------------------------------------------

dataset: 1
target: OK091006.1
length: 29836
miRNA : 5027
length: 31

mfe: -31.0 kcal/mol
p-value: 1.000000e+00

position  6131
target 5' G                 GAUUAUAA       A      3'
           GUG AUGUGGUGGCUAUU        ACACUAC
           CGC UGCACUAUUGGUGA        UGUGAUG
miRNA  3'     U                            CCUUUG 5'

------------------------------------------------------------

dataset: 1
target: OK091006.1
length: 29836
miRNA : 5027
```

```
length: 31

mfe: -30.0 kcal/mol
p-value: 1.000000e+00

position  6426
target 5' A   U       A      CGAAG UG         A 3'
           GUG AAUGUGA AACUAC     U  UA GGAGAC
           CGC UUGCACU UUGGUG     G  AU CCUUUG
miRNA  3'             A      AU    UG  G        5'

------------------------------------------------------------

dataset: 1
target: OK091006.1
length: 29836
miRNA : 5027
length: 31

mfe: -29.9 kcal/mol
p-value: 1.000000e+00

position  29226
target 5' C G        U            A    U   C 3'
           G GAACGUGG UGACC   UACAC  GG GC
           C CUUGCACU AUUGG   AUGUG  CC UG
miRNA  3'   G              UG     AUG  UU    5'

------------------------------------------------------------

dataset: 1
target: OK091006.1
length: 29836
miRNA : 5027
length: 31

mfe: -29.6 kcal/mol
p-value: 1.000000e+00

position  12210
target 5' U   C       CA     GCA  GUAAGU      A 3'
           UGA CGUGAUG  GCCAU   AC      UGGAAA
           GCU GCACUAU  UGGUG   UG      GCCUUU
miRNA  3' C   U              AUG  AU          G 5'

------------------------------------------------------------

dataset: 1
target: OK091006.1
length: 29836
miRNA : 5027
length: 31

mfe: -29.3 kcal/mol
p-value: 1.000000e+00

position  10090
```

```
target 5'     U       CA         UA    UCUUU   U 3'
               UGUGGUA   ACUACACU  ACGG     GGC
               GCACUAU   UGAUGUGA  UGCC     UUG
miRNA  3' CGCUU       UGG              U         5'
```

----

dataset: 1
target: OK091006.1
length: 29836
miRNA : 5027
length: 31

mfe: -29.2 kcal/mol
p-value: 1.000000e+00

```
position  10641
target 5'   U   U    UGUA      UG UAUAA        A 3'
             AGC UGGU    CGCUGC  U     AUGGAGAC
             UUG ACUA    GUGAUG  A     UGCCUUUG
miRNA  3' CGC   C    UUG       UG                5'
```

----

dataset: 1
target: OK091006.1
length: 29836
miRNA : 5027
length: 31

mfe: -29.0 kcal/mol
p-value: 1.000000e+00

```
position  1430
target 5' C  A  G     CGC           UU      U 3'
           GU AG GUGGU   ACUAUUGC CU  GGAGGC
           CG UU CACUA   UGGUGAUG GA  CCUUUG
miRNA  3'    C  G     U          U  UG        5'
```

----

dataset: 1
target: OK091006.1
length: 29836
miRNA : 5027
length: 31

mfe: -29.0 kcal/mol
p-value: 1.000000e+00

```
position  8753
target 5' A  C        GG AG        AU  CAA   U 3'
           GC AGCGUGGU  U  UUAUACUA  GA   AGC
           CG UUGCACUA  G  GAUGUGAU  CU   UUG
miRNA  3'    C        UU GU        GC          5'
```

```
dataset: 1
target: OK091006.1
length: 29836
miRNA : 5027
length: 31

mfe: -29.0 kcal/mol
p-value: 1.000000e+00

position  29069
target 5' G  A             GA   AACCCAA      U 3'
           GC GACGUGGU  CCA  ACA       GGAAAU
           CG UUGCACUA  GGU  UGU       CCUUUG
miRNA  3'    C        UU   GA   GAUG           5'

------------------------------------------------------------

dataset: 1
target: OK091006.1
length: 29836
miRNA : 5027
length: 31

mfe: -28.9 kcal/mol
p-value: 1.000000e+00

position  29677
target 5'  A    U   AAG      C     UUC  C     C 3'
            GGAC UGA   AGCCAC ACAUU   AC GAGGC
            CUUG ACU   UUGGUG UGUGA   UG CUUUG
miRNA  3' CG    C   A        A          C       5'

------------------------------------------------------------

dataset: 1
target: OK091006.1
length: 29836
miRNA : 5027
length: 31

mfe: -28.7 kcal/mol
p-value: 1.000000e+00

position  6339
target 5' A G                        A    C 3'
           G GAAUG GAUAAUC UUGC CUGCG AGAU
           C CUUGC CUAUUGG GAUG GAUGC UUUG
miRNA  3'   G     A       U    U     C      5'

------------------------------------------------------------

dataset: 1
target: OK091006.1
length: 29836
miRNA : 5027
length: 31
```

```
mfe: -28.6 kcal/mol
p-value: 1.000000e+00

position  4131
target 5'     C      UAU   U   AAAAAGUCUGGUG       U     G 3'
               UGUGGU   ACC ACU             GCACUAC GAAAU
               GCACUA   UGG UGA             UGUGAUG CUUUG
miRNA  3' CGCUU      U                             C       5'

------------------------------------------------------------

dataset: 1
target: OK091006.1
length: 29836
miRNA : 5027
length: 31

mfe: -28.4 kcal/mol
p-value: 1.000000e+00

position  27046
target 5' C         UAGCAG      CAGGUUUU     AUACAGUC     A   UU   A 3'
           GC AGCGUG      GUGACU        GCUGC        GCUAC GGA  GGC
           CG UUGCAC      UAUUGG        UGAUG        UGAUG CCU  UUG
miRNA  3'    C                                                       5'

------------------------------------------------------------

dataset: 1
target: OK091006.1
length: 29836
miRNA : 5027
length: 31

mfe: -28.0 kcal/mol
p-value: 1.000000e+00

position  2219
target 5' G      AAAU  UC    UG    AA        U 3'
           GUGGAC    UG   ACC  UGCA    GGAAAU
           CGCUUG    AC   UGG  AUGU    CCUUUG
miRNA  3'        C     UAU   UG    GAUG        5'

------------------------------------------------------------

dataset: 1
target: OK091006.1
length: 29836
miRNA : 5027
length: 31

mfe: -27.8 kcal/mol
p-value: 1.000000e+00

position  8673
target 5' U   UG            CGUGACAUA   U    A  U  U 3'
```

```
           UGA  GUGGUG UCACU         GCA CUAC GA AC
           GCU  CACUAU GGUGA         UGU GAUG CU UG
miRNA  3' C   UG      U                     C  U    5'
```

----

dataset: 1
target: OK091006.1
length: 29836
miRNA : 5027
length: 31

mfe: -27.7 kcal/mol
p-value: 1.000000e+00

```
position  626
target 5'  G              A         C    3'
            GAGC UGGUGGCCAU   GUUACGG
            CUUG ACUAUUGGUG   UGAUGCC
miRNA  3' CG    C          AUG       UUUG 5'
```

----

dataset: 1
target: OK091006.1
length: 29836
miRNA : 5027
length: 31

mfe: -27.7 kcal/mol
p-value: 1.000000e+00

```
position  28565
target 5'  A  UG     UU               C  3'
            GA  GU AU  CUACUAC CUA GGAA
            CU  CA UA  GGUGAUG GAU CCUU
miRNA  3' CG  UG  C  UU       U   G    UG 5'
```

----

dataset: 1
target: OK091006.1
length: 29836
miRNA : 5027
length: 31

mfe: -27.7 kcal/mol
p-value: 1.000000e+00

```
position  17130
target 5' A  UU  UCUCAU      GUUGAU        U    A 3'
           GC  GC      GCCGCU      GCACUAUG GAGA
           CG  UG      UGGUGA      UGUGAUGC CUUU
miRNA  3'    CU  CACUAU                        G 5'
```

```
dataset: 1
target: OK091006.1
length: 29836
miRNA : 5027
length: 31

mfe: -27.7 kcal/mol
p-value: 1.000000e+00

position  19816
target 5' U  GG     ACAUU      A      UGAUC     CU  A 3'
           UG  UGUGG     GCUGCU AUACUG     UGGGA  AC
           GC  GCACU     UGGUGA UGUGAU     GCCUU  UG
miRNA  3' C  UU     AU                              5'

------------------------------------------------------------

dataset: 1
target: OK091006.1
length: 29836
miRNA : 5027
length: 31

mfe: -27.7 kcal/mol
p-value: 1.000000e+00

position  749
target 5' C    ACUCAU      GCU    GGAGGGGCAUA           UC   A 3'
           GUGA      GCGUGA   UAAC           CACU CGCUAUG  GAU
           CGCU      UGCACU   AUUG           GUGA GUGAUGC  UUG
miRNA  3'                                        U       CU     5'

------------------------------------------------------------

dataset: 1
target: OK091006.1
length: 29836
miRNA : 5027
length: 31

mfe: -27.6 kcal/mol
p-value: 1.000000e+00

position  25904
target 5' C     A   CU    GAUUGGUGG       GAAAA        C 3'
           UGAAC UGA  ACCA         UUAUACU     AUGGGAAU
           GCUUG ACU  UGGU         GAUGUGA     UGCCUUUG
miRNA  3' C     C   AU                                 5'

------------------------------------------------------------

dataset: 1
target: OK091006.1
length: 29836
miRNA : 5027
length: 31
```

```
mfe: -27.4 kcal/mol
p-value: 1.000000e+00

position  3492
target 5' U     C          AUA     UAAU   C   3'
           UGAAU UGAUGAUUAC   GCUAC    GGA
           GCUUG ACUAUUGGUG   UGAUG    CCU
miRNA  3' C     C          AUG            UUG 5'

------------------------------------------------------------

dataset: 1
target: OK091006.1
length: 29836
miRNA : 5027
length: 31

mfe: -27.4 kcal/mol
p-value: 1.000000e+00

position  4563
target 5' A    ACUCU   UACAAU      UG      U    A 3'
           UGAA     UGU      GCCACU  GCUAUG  AAC
           GCUU     GCA      UGGUGA  UGAUGC  UUG
miRNA  3' C            CUAU        UG      CU     5'

------------------------------------------------------------

dataset: 1
target: OK091006.1
length: 29836
miRNA : 5027
length: 31

mfe: -27.3 kcal/mol
p-value: 1.000000e+00

position  308
target 5' C         CUCG    GUG   UU      U 3'
           GCG ACGUG     UAC   GCU  GGAGAC
           CGC UGCAC     GUG   UGA  CCUUUG
miRNA  3'     U     UAUUG   AUG   UG        5'

------------------------------------------------------------

dataset: 1
target: OK091006.1
length: 29836
miRNA : 5027
length: 31

mfe: -27.3 kcal/mol
p-value: 1.000000e+00

position  1911
target 5' C  U     UUUUACAGAA         AACAAU   AG      U  3'
           UG GCGUG          GGCCGCUAU      ACU  AUGGAA
```

```
           GC UGCAC          UUGGUGAUG      UGA  UGCCUU
miRNA  3' C  U     UA                                  UG 5'

------------------------------------------------------------
```

Delta Top 25 tDR-Gly

```
ersion: RNAhybrid 2.2
Command line:/vol/bioapps/bin/RNAhybrid.bin -s 3utr_human -m 29836 -t
/var/bibiserv2/anonymous/rnahybrid/28/15/39/bibiserv2_2022-06-
28_153936_UU6f6/rnahybrid_input_target_rna_sequences_.file -n 30 -q
/var/bibiserv2/anonymous/rnahybrid/28/15/39/bibiserv2_2022-06-
28_153936_UU6f6/rnahybrid_input_mirna_sequences.file -b 25
searching
dataset: 1
mde of 5008c: -65.000008
Individual hits
------------------------------------------------------------

dataset: 1
target: OK091006.1
length: 29836
miRNA : 5008c
length: 30

mfe: -35.7 kcal/mol
p-value: 1.000000e+00

position  1459
target 5' A        GUUCUCUUAUGUUGG       AACAAG      UAUUGGGUUCCACGU        U
3'
           GGCUGUGU               UUGCCAU      UGUGCC               GCUAGCGC
           UCGAUACG               AGUGGUG      AUAUGG               UGGUUGCG
miRNA  3' G
5'

------------------------------------------------------------

dataset: 1
target: OK091006.1
length: 29836
miRNA : 5008c
length: 30

mfe: -35.3 kcal/mol
p-value: 1.000000e+00

position  10370
target 5' U     UGUUACAAUG        U   GUGU          G 3'
           UAGCU          GUUCACCA CUG    UUACCAAUGU
           GUCGA          CGAGUGGU GAU    GGUGGUUGCG
miRNA  3'       UA                    AU              5'

------------------------------------------------------------

dataset: 1
target: OK091006.1
```

```
length: 29836
miRNA : 5008c
length: 30

mfe: -35.2 kcal/mol
p-value: 1.000000e+00

position  17128
target 5' A                         GUU     A 3'
           CAGCU UGCUC UCAU   GCCGCU   GAUGC
           GUCGA ACGAG GGUG   UGGUGG   UUGCG
miRNA  3'       U     U    AUA                5'

------------------------------------------------------------

dataset: 1
target: OK091006.1
length: 29836
miRNA : 5008c
length: 30

mfe: -33.0 kcal/mol
p-value: 1.000000e+00

position  14547
target 5'  U       A       UCUGGUAAUC        GAUAA    A 3'
            GCUAUGC CGCUGCU          UAUUACUA     ACGC
            CGAUACG GUGGUGA          AUGGUGGU     UGCG
miRNA  3' GU       A       U                            5'

------------------------------------------------------------

dataset: 1
target: OK091006.1
length: 29836
miRNA : 5008c
length: 30

mfe: -32.9 kcal/mol
p-value: 1.000000e+00

position  13733
target 5' A    GAC   G       A        GU      C 3'
           CGGU   AUG  UACCAC UAUAUCAC  CAACGU
           GUCG   UAC  GUGGUG AUAUGGUG  GUUGCG
miRNA  3'      A     GA                         5'

------------------------------------------------------------

dataset: 1
target: OK091006.1
length: 29836
miRNA : 5008c
length: 30

mfe: -32.7 kcal/mol
p-value: 1.000000e+00
```

```
position  17406
target 5' U   GACCC      AU        UG  CC    A 3'
           GGC     UGCUCA      UACC  CA  ACGC
           UCG     ACGAGU      AUGG  GU  UGCG
miRNA  3' G   AU         GGUGAU    UG          5'

------------------------------------------------------------

dataset: 1
target: OK091006.1
length: 29836
miRNA : 5008c
length: 30

mfe: -32.5 kcal/mol
p-value: 1.000000e+00

position  28393
target 5'  U  GUCUUG          CUC  U  A  UG  A 3'
            GC      GUUCACCGCU   AC CA CA  GC
            CG      CGAGUGGUGA   UG GU GU  CG
miRNA  3' GU  AUA             UA      G  UG    5'

------------------------------------------------------------

dataset: 1
target: OK091006.1
length: 29836
miRNA : 5008c
length: 30

mfe: -32.4 kcal/mol
p-value: 1.000000e+00

position  29479
target 5' G   A     G   CAACUCAGGCCUAA   CAUGCAG     A   G  A 3'
           AGC GUGCU ACU              ACU       ACCAC CAA GC
           UCG UACGA UGG              UGA       UGGUG GUU CG
miRNA  3' G   A     G                    UA              G    5'

------------------------------------------------------------

dataset: 1
target: OK091006.1
length: 29836
miRNA : 5008c
length: 30

mfe: -32.3 kcal/mol
p-value: 1.000000e+00

position  26679
target 5' U     UGUUUU              UU  AGAAUAAAUUGGAU    GG  G 3'
           UAGCU      GUGCUUGCUGCUGU  AC              CACC  UG
           GUCGA      UACGAGUGGUGAUA  UG              GUGG  GC
miRNA  3'                                                 UU  G 5'
```

```
------------------------------------------------------------

dataset: 1
target: OK091006.1
length: 29836
miRNA : 5008c
length: 30

mfe: -32.3 kcal/mol
p-value: 1.000000e+00

position  24199
target 5' U   GCAG     G      AA        UGCU    A 3'
           GGU    GUGCU CAUUAC  AUACCAUU    AUGC
           UCG    UACGA GUGGUG  UAUGGUGG    UGCG
miRNA  3' G   A               A         U         5'

------------------------------------------------------------

dataset: 1
target: OK091006.1
length: 29836
miRNA : 5008c
length: 30

mfe: -32.0 kcal/mol
p-value: 1.000000e+00

position  4652
target 5' C      CAGUUUC   UUCU     UG      GUUA      A 3'
           CAGCUA       UGU    UCACC   AUGCU    CAGCGU
           GUCGAU       ACG    AGUGG   UAUGG    GUUGCG
miRNA  3'                          UGA     UG          5'

------------------------------------------------------------

dataset: 1
target: OK091006.1
length: 29836
miRNA : 5008c
length: 30

mfe: -32.0 kcal/mol
p-value: 1.000000e+00

position  26773
target 5' U      CUUCAU        UUCAG    U UG   GU    G 3'
           CAGCUA      UGCUU CU     ACUG U  CGC  ACGC
           GUCGAU      ACGAG GG     UGAU A  GUG  UGCG
miRNA  3'                  U             UG   GU      5'

------------------------------------------------------------

dataset: 1
target: OK091006.1
length: 29836
```

```
miRNA : 5008c
length: 30

mfe: -31.7 kcal/mol
p-value: 1.000000e+00

position  16900
target 5' A    A    CAUUAAGUG    UAC    G      AAGA  A 3'
           CAGU AUGC         CACC   ACUA UGCCAC    GC
           GUCG UACG         GUGG   UGAU AUGGUG    CG
miRNA  3'      A    A                          GUUG    5'

------------------------------------------------------------

dataset: 1
target: OK091006.1
length: 29836
miRNA : 5008c
length: 30

mfe: -31.6 kcal/mol
p-value: 1.000000e+00

position  1290
target 5' A  AAG            UGUGGUUACU        AA    U 3'
           AG   GUGC CACUACU          UACC CCA  AUGC
           UC   UACG GUGGUGA          AUGG GGU  UGCG
miRNA  3' G  GA     A       U             U           5'

------------------------------------------------------------

dataset: 1
target: OK091006.1
length: 29836
miRNA : 5008c
length: 30

mfe: -31.6 kcal/mol
p-value: 1.000000e+00

position  29147
target 5' U   C    AAA UG   AAUU    C       U 3'
           GGC   GC   U  CAC    UGCC CCAGCGC
           UCG   CG   G  GUG    AUGG GGUUGCG
miRNA  3' G   AUA  A   UG   AU      U         5'

------------------------------------------------------------

dataset: 1
target: OK091006.1
length: 29836
miRNA : 5008c
length: 30

mfe: -31.5 kcal/mol
p-value: 1.000000e+00
```

```
position  26967
target 5'  C    UGACAUCAAGGACC   CUAAAGAAA     GUU    C     G     U 3'
            GCUG              UGC          UCACU   GCUA AUCAC AACGC
            CGAU              ACG          AGUGG   UGAU UGGUG UUGCG
miRNA  3' GU                                        A     G       5'
```

----

dataset: 1
target: OK091006.1
length: 29836
miRNA : 5008c
length: 30

mfe: -31.5 kcal/mol
p-value: 1.000000e+00

```
position  14762
target 5' U    G  UA G        CGUUAUAAU        A  3'
           CAGC AU  U ACUACUAU         CUACCAAC
           GUCG UA  A UGGUGAUA         GGUGGUUG
miRNA  3'      A  CG G        U                CG 5'
```

----

dataset: 1
target: OK091006.1
length: 29836
miRNA : 5008c
length: 30

mfe: -31.4 kcal/mol
p-value: 1.000000e+00

```
position  16178
target 5' A        ACA            AGU  UAC G  U 3'
           GGCUAUGU   CACCGC AUAC   CU   A GC
           UCGAUACG   GUGGUG UAUG   GG   U CG
miRNA  3' G        A        A    GU   U   G    5'
```

----

dataset: 1
target: OK091006.1
length: 29836
miRNA : 5008c
length: 30

mfe: -31.4 kcal/mol
p-value: 1.000000e+00

```
position  29689
target 5'       A         AUUU       G  C 3'
                 GC CACCAC    UCACCGA GC
                 CG GUGGUG    GGUGGUU CG
miRNA  3' GUCGAUA  A      AUAU       G    5'
```

```
------------------------------------------------------------

dataset: 1
target: OK091006.1
length: 29836
miRNA : 5008c
length: 30

mfe: -31.3 kcal/mol
p-value: 1.000000e+00

position  11080
target 5'  U      GGUA           U     UU   A 3'
            GCUAUG    UUAUUGCUAUG CUGCU  UGC
            CGAUAC    AGUGGUGAUAU GGUGG  GCG
miRNA  3' GU      G                   UU     5'

------------------------------------------------------------

dataset: 1
target: OK091006.1
length: 29836
miRNA : 5008c
length: 30

mfe: -31.2 kcal/mol
p-value: 1.000000e+00

position  13201
target 5' U   G    A   UGU GU           U   C 3'
           GGU GUGC UCG   U  CUGUACUGCCG UGC
           UCG UACG AGU   G  GAUAUGGUGGU GCG
miRNA  3' G   A            GU           U     5'

------------------------------------------------------------

dataset: 1
target: OK091006.1
length: 29836
miRNA : 5008c
length: 30

mfe: -31.1 kcal/mol
p-value: 1.000000e+00

position  1151
target 5' C         G      AAUGA    A     AUG   C 3'
           CAGU  UGC UCACCA     AUGC ACCAA   UGC
           GUCG  ACG AGUGGU     UAUG UGGUU   GCG
miRNA  3'      AU           GA       G             5'

------------------------------------------------------------

dataset: 1
target: OK091006.1
length: 29836
miRNA : 5008c
```

```
length: 30

mfe: -31.1 kcal/mol
p-value: 1.000000e+00

position  2838
target 5' A   AAA  A       UG   UG  G  G    U 3'
           AGU   UG GUUCGCC  UGU  UG CA AUGC
           UCG   AU CGAGUGG  AUA  GU GU UGCG
miRNA  3' G        A       UG   UG  G         5'

------------------------------------------------------------

dataset: 1
target: OK091006.1
length: 29836
miRNA : 5008c
length: 30

mfe: -30.9 kcal/mol
p-value: 1.000000e+00

position  18206
target 5' A    A   G         ACAUGUU       C   A 3'
           AGUU AUG UUACC CUA       UAUCACC GCG
           UCGA UAC AGUGG GAU       AUGGUGG UGC
miRNA  3' G        G     U                 U   G 5'

------------------------------------------------------------

dataset: 1
target: OK091006.1
length: 29836
miRNA : 5008c
length: 30

mfe: -30.9 kcal/mol
p-value: 1.000000e+00

position  25226
target 5' A  A        UG UG   G    G      U 3'
           CA UUAUGCUU  C  UAU ACCA U  UGC
           GU GAUACGAG  G  AUA UGGU G  GCG
miRNA  3'    C        UG UG        G UU     5'

------------------------------------------------------------
```

BA.1 top 25 with tDR-Val

```
Version: RNAhybrid 2.2
Command line:/vol/bioapps/bin/RNAhybrid.bin -n 31 -m 29684 -b 25 -s
3utr_human -q /var/bibiserv2/anonymous/rnahybrid/28/15/23/bibiserv2_2022-06-
28_152328_Bgqvy/rnahybrid_input_mirna_sequences.file -t
/var/bibiserv2/anonymous/rnahybrid/28/15/23/bibiserv2_2022-06-
28_152328_Bgqvy/rnahybrid_input_target_rna_sequences_.file
```

```
searching
dataset: 1
mde of 5027: -64.200005
Individual hits
------------------------------------------------------------

dataset: 1
target: OL672836.1
length: 29684
miRNA : 5027
length: 31

mfe: -31.0 kcal/mol
p-value: 1.000000e+00

position  6104
target 5' G                  GAUUAUAA       A      3'
           GUG AUGUGGUGGCUAUU        ACACUAC
           CGC UGCACUAUUGGUGA        UGUGAUG
miRNA  3'     U                             CCUUUG 5'

------------------------------------------------------------

dataset: 1
target: OL672836.1
length: 29684
miRNA : 5027
length: 31

mfe: -30.9 kcal/mol
p-value: 1.000000e+00

position  29620
target 5'  A    U   AAG      C     UUCACCGAGGCCAC      U  G 3'
            GGAC UGA   AGCCAC ACAUU              GCGGAG AC
            CUUG ACU   UUGGUG UGUGA              UGCCUU UG
miRNA  3' CG    C   A        A                              5'

------------------------------------------------------------

dataset: 1
target: OL672836.1
length: 29684
miRNA : 5027
length: 31

mfe: -30.5 kcal/mol
p-value: 1.000000e+00

position  5453
target 5' U           UGUAAA   UG  G  A  A C    A 3'
           UGAACGUGGUG      ACU  UG AC AC G AGAC
           GCUUGCACUAU      UGG  AU UG UG C UUUG
miRNA  3' C                    UG  G  A  C        5'

------------------------------------------------------------
```

```
dataset: 1
target: OL672836.1
length: 29684
miRNA : 5027
length: 31

mfe: -30.0 kcal/mol
p-value: 1.000000e+00

position  6399
target 5' A   U       A      CGAAG UG         A 3'
           GUG AAUGUGA AACUAC     U  UA GGAGAC
           CGC UUGCACU UUGGUG     G  AU CCUUUG
miRNA  3'             A      AU    UG  G        5'

------------------------------------------------------------

dataset: 1
target: OL672836.1
length: 29684
miRNA : 5027
length: 31

mfe: -29.9 kcal/mol
p-value: 1.000000e+00

position  29169
target 5' C G        U            A    U   C 3'
           G GAACGUGG UGACC  UACAC   GG  GC
           C CUUGCACU AUUGG  AUGUG   CC  UG
miRNA  3'   G              UG     AUG  UU    5'

------------------------------------------------------------

dataset: 1
target: OL672836.1
length: 29684
miRNA : 5027
length: 31

mfe: -29.6 kcal/mol
p-value: 1.000000e+00

position  12171
target 5' U   C       CA     GCA  GUAAGU      A 3'
           UGA CGUGAUG  GCCAU   AC      UGGAAA
           GCU GCACUAU  UGGUG   UG      GCCUUU
miRNA  3' C   U              AUG  AU          G 5'

------------------------------------------------------------

dataset: 1
target: OL672836.1
length: 29684
miRNA : 5027
length: 31
```

mfe: -29.3 kcal/mol
p-value: 1.000000e+00

```
position  10060
target 5'     U       CA         UA    UCUUU   U 3'
               UGUGGUA   ACUACACU  ACGG     GGC
               GCACUAU   UGAUGUGA  UGCC     UUG
miRNA  3' CGCUU       UGG              U         5'
```

----

dataset: 1
target: OL672836.1
length: 29684
miRNA : 5027
length: 31

mfe: -29.2 kcal/mol
p-value: 1.000000e+00

```
position  10611
target 5'   U   U    UGUA      UG UAUAA        A 3'
             AGC UGGU    CGCUGC  U     AUGGAGAC
             UUG ACUA    GUGAUG  A     UGCCUUUG
miRNA  3' CGC   C    UUG       UG                5'
```

----

dataset: 1
target: OL672836.1
length: 29684
miRNA : 5027
length: 31

mfe: -29.0 kcal/mol
p-value: 1.000000e+00

```
position  1403
target 5' C  A  G     CGC          UU      U 3'
           GU AG GUGGU   ACUAUUGC CU  GGAGGC
           CG UU CACUA   UGGUGAUG GA  CCUUUG
miRNA  3'    C  G     U          U  UG        5'
```

----

dataset: 1
target: OL672836.1
length: 29684
miRNA : 5027
length: 31

mfe: -29.0 kcal/mol
p-value: 1.000000e+00

```
position  8723
target 5' A  C        GG AG        AU  CAA   U 3'
           GC AGCGUGGU  U  UUAUACUA  GA   AGC
```

```
           CG UUGCACUA  G  GAUGUGAU  CU   UUG
miRNA  3'    C        UU GU        GC          5'
```

----

dataset: 1
target: OL672836.1
length: 29684
miRNA : 5027
length: 31

mfe: -29.0 kcal/mol
p-value: 1.000000e+00

position 29012

```
target 5' G  A             GA   AACCCAA      U 3'
           GC GACGUGGU  CCA  ACA       GGAAAU
           CG UUGCACUA  GGU  UGU       CCUUUG
miRNA  3'    C        UU   GA   GAUG           5'
```

----

dataset: 1
target: OL672836.1
length: 29684
miRNA : 5027
length: 31

mfe: -28.7 kcal/mol
p-value: 1.000000e+00

position 6312

```
target 5' A G                        A    C 3'
           G GAAUG GAUAAUC UUGC CUGCG AGAU
           C CUUGC CUAUUGG GAUG GAUGC UUUG
miRNA  3'   G     A       U    U     C      5'
```

----

dataset: 1
target: OL672836.1
length: 29684
miRNA : 5027
length: 31

mfe: -28.6 kcal/mol
p-value: 1.000000e+00

position 4104

```
target 5'    C      UAU   U   AAAAAGGCUGGUG       U     G 3'
               UGUGGU   ACC ACU             GCACUAC GAAAU
               GCACUA   UGG UGA             UGUGAUG CUUUG
miRNA  3' CGCUU      U                            C       5'
```

----

dataset: 1

target: OL672836.1
length: 29684
miRNA : 5027
length: 31

mfe: -28.4 kcal/mol
p-value: 1.000000e+00

```
position  26998
target 5' C         UAGCAG      CAGGUUUU     AUACAGUC     A   UU   A 3'
           GC AGCGUG      GUGACU        GCUGC        GCUAC GGA  GGC
           CG UUGCAC      UAUUGG        UGAUG        UGAUG CCU  UUG
miRNA  3'    C                                                     5'
```

----

dataset: 1
target: OL672836.1
length: 29684
miRNA : 5027
length: 31

mfe: -28.0 kcal/mol
p-value: 1.000000e+00

```
position  2192
target 5' G      AAAU  UC    UG    AA        U 3'
           GUGGAC    UG   ACC  UGCA    GGAAAU
           CGCUUG    AC   UGG  AUGU    CCUUUG
miRNA  3'       C     UAU   UG    GAUG        5'
```

----

dataset: 1
target: OL672836.1
length: 29684
miRNA : 5027
length: 31

mfe: -27.8 kcal/mol
p-value: 1.000000e+00

```
position  8643
target 5' U   UG            CGUGACAUA   U    A  U  U 3'
           UGA  GUGGUG UCACU         GCA CUAC GA AC
           GCU  CACUAU GGUGA         UGU GAUG CU UG
miRNA  3' C   UG      U                     C  U    5'
```

----

dataset: 1
target: OL672836.1
length: 29684
miRNA : 5027
length: 31

mfe: -27.7 kcal/mol

```
p-value: 1.000000e+00

position  599
target 5'  G               A         C    3'
            GAGC UGGUGGCCAU   GUUACGG
            CUUG ACUAUUGGUG   UGAUGCC
miRNA  3' CG    C          AUG       UUUG 5'

------------------------------------------------------------

dataset: 1
target: OL672836.1
length: 29684
miRNA : 5027
length: 31

mfe: -27.7 kcal/mol
p-value: 1.000000e+00

position  28508
target 5'  A  UG     UU                C  3'
            GA  GU AU  CUACUAC CUA GGAA
            CU  CA UA  GGUGAUG GAU CCUU
miRNA  3' CG  UG  C  UU       U   G    UG 5'

------------------------------------------------------------

dataset: 1
target: OL672836.1
length: 29684
miRNA : 5027
length: 31

mfe: -27.7 kcal/mol
p-value: 1.000000e+00

position  17091
target 5' A  UU  UCUCAU      GUUGAU        U    A 3'
           GC  GC      GCCGCU      GCACUAUG GAGA
           CG  UG      UGGUGA      UGUGAUGC CUUU
miRNA  3'    CU  CACUAU                         G 5'

------------------------------------------------------------

dataset: 1
target: OL672836.1
length: 29684
miRNA : 5027
length: 31

mfe: -27.7 kcal/mol
p-value: 1.000000e+00

position  19777
target 5' U  GG     ACAUU      A      UGAUC     CU  A 3'
           UG  UGUGG     GCUGCU AUACUG     UGGGA  AC
           GC  GCACU     UGGUGA UGUGAU     GCCUU  UG
```

```
miRNA  3' C  UU     AU                                5'

------------------------------------------------------------

dataset: 1
target: OL672836.1
length: 29684
miRNA : 5027
length: 31

mfe: -27.7 kcal/mol
p-value: 1.000000e+00

position  722
target 5' C    ACUCAU      GCU    GGAGGGGCAUA            UC   A 3'
           GUGA      GCGUGA   UAAC           CACU CGCUAUG  GAU
           CGCU      UGCACU   AUUG           GUGA GUGAUGC  UUG
miRNA  3'                                        U       CU     5'

------------------------------------------------------------

dataset: 1
target: OL672836.1
length: 29684
miRNA : 5027
length: 31

mfe: -27.6 kcal/mol
p-value: 1.000000e+00

position  25856
target 5' C   A     CU    GAUUGGUGG       GAAAA        C 3'
           UGAAC UGA  ACCA         UUAUACU     AUGGGAAU
           GCUUG ACU  UGGU         GAUGUGA     UGCCUUUG
miRNA  3' C     C   AU                                   5'

------------------------------------------------------------

dataset: 1
target: OL672836.1
length: 29684
miRNA : 5027
length: 31

mfe: -27.4 kcal/mol
p-value: 1.000000e+00

position  3465
target 5' U     C          AUA     UAAU   C   3'
           UGAAU UGAUGAUUAC   GCUAC    GGA
           GCUUG ACUAUUGGUG   UGAUG    CCU
miRNA  3' C     C          AUG            UUG 5'

------------------------------------------------------------

dataset: 1
target: OL672836.1
```

```
length: 29684
miRNA : 5027
length: 31

mfe: -27.4 kcal/mol
p-value: 1.000000e+00

position  4536
target 5' A    ACUCU   UACAAU      UG      U    A 3'
           UGAA     UGU      GCCACU  GCUAUG  AAC
           GCUU     GCA      UGGUGA  UGAUGC  UUG
miRNA  3' C            CUAU        UG      CU     5'

------------------------------------------------------------

dataset: 1
target: OL672836.1
length: 29684
miRNA : 5027
length: 31

mfe: -27.3 kcal/mol
p-value: 1.000000e+00

position  281
target 5' C         CUCG    GUG   UU      U 3'
           GCG ACGUG     UAC   GCU  GGAGAC
           CGC UGCAC     GUG   UGA  CCUUUG
miRNA  3'     U     UAUUG   AUG   UG        5'

------------------------------------------------------------
```

BA.1 top 25 with tDR-Gly

```
Version: RNAhybrid 2.2
Command line:/vol/bioapps/bin/RNAhybrid.bin -n 30 -b 25 -m 29684 -q
/var/bibiserv2/anonymous/rnahybrid/28/15/27/bibiserv2_2022-06-
28_152716_cgBUf/rnahybrid_input_mirna_sequences.file -s 3utr_human -t
/var/bibiserv2/anonymous/rnahybrid/28/15/27/bibiserv2_2022-06-
28_152716_cgBUf/rnahybrid_input_target_rna_sequences_.file
searching
dataset: 1
mde of 5008c: -65.000008
Individual hits
------------------------------------------------------------

dataset: 1
target: OL672836.1
length: 29684
miRNA : 5008c
length: 30

mfe: -35.7 kcal/mol
p-value: 1.000000e+00

position  1432
```

```
target 5' A        GUUCUCUUAUGUUGG       AACAAG      UAUUGGGUUCCACGU        U
3'
           GGCUGUGU               UUGCCAU      UGUGCC               GCUAGCGC
           UCGAUACG               AGUGGUG      AUAUGG               UGGUUGCG
miRNA  3' G
5'

------------------------------------------------------------

dataset: 1
target: OL672836.1
length: 29684
miRNA : 5008c
length: 30

mfe: -35.3 kcal/mol
p-value: 1.000000e+00

position  10340
target 5' U     UGUUACAAUG        U   GUGU           G 3'
           UAGCU          GUUCACCA CUG    UUACCAAUGU
           GUCGA          CGAGUGGU GAU    GGUGGUUGCG
miRNA  3'       UA                   AU               5'

------------------------------------------------------------

dataset: 1
target: OL672836.1
length: 29684
miRNA : 5008c
length: 30

mfe: -35.2 kcal/mol
p-value: 1.000000e+00

position  17089
target 5' A                         GUU     A 3'
           CAGCU UGCUC UCAU   GCCGCU   GAUGC
           GUCGA ACGAG GGUG   UGGUGG   UUGCG
miRNA  3'       U     U    AUA                5'

------------------------------------------------------------

dataset: 1
target: OL672836.1
length: 29684
miRNA : 5008c
length: 30

mfe: -33.0 kcal/mol
p-value: 1.000000e+00

position  14508
target 5'  U       A       UCUGGUAAUC        GAUAA    A 3'
            GCUAUGC CGCUGCU          UAUUACUA     ACGC
            CGAUACG GUGGUGA          AUGGUGGU     UGCG
miRNA  3' GU       A       U                           5'
```

```
------------------------------------------------------------

dataset: 1
target: OL672836.1
length: 29684
miRNA : 5008c
length: 30

mfe: -32.9 kcal/mol
p-value: 1.000000e+00

position  13694
target 5' A    GAC   G       A        GU      C 3'
           CGGU   AUG  UACCAC UAUAUCAC  CAACGU
           GUCG   UAC  GUGGUG AUAUGGUG  GUUGCG
miRNA  3'      A     GA                         5'

------------------------------------------------------------

dataset: 1
target: OL672836.1
length: 29684
miRNA : 5008c
length: 30

mfe: -32.7 kcal/mol
p-value: 1.000000e+00

position  17367
target 5' U   GACCC      AU        UG  CC    A 3'
           GGC     UGCUCA      UACC  CA  ACGC
           UCG     ACGAGU      AUGG  GU  UGCG
miRNA  3' G   AU         GGUGAU    UG          5'

------------------------------------------------------------

dataset: 1
target: OL672836.1
length: 29684
miRNA : 5008c
length: 30

mfe: -32.5 kcal/mol
p-value: 1.000000e+00

position  28336
target 5'  U  GUCUUG          CUC  U  A  UG  A 3'
            GC      GUUCACCGCU   AC CA CA  GC
            CG      CGAGUGGUGA   UG GU GU  CG
miRNA  3' GU  AUA             UA      G  UG    5'

------------------------------------------------------------

dataset: 1
target: OL672836.1
length: 29684
```

```
miRNA : 5008c
length: 30

mfe: -32.4 kcal/mol
p-value: 1.000000e+00

position  29422
target 5' G   A     G   CAACUCAGGCCUAA   CAUGCAG     A   G  A 3'
           AGC GUGCU ACU              ACU       ACCAC CAA GC
           UCG UACGA UGG              UGA       UGGUG GUU CG
miRNA  3' G   A     G                    UA              G    5'

------------------------------------------------------------

dataset: 1
target: OL672836.1
length: 29684
miRNA : 5008c
length: 30

mfe: -32.3 kcal/mol
p-value: 1.000000e+00

position  24151
target 5' U   GCAG     G      AA        UGCU    A 3'
           GGU    GUGCU CAUUAC  AUACCAUU    AUGC
           UCG    UACGA GUGGUG  UAUGGUGG    UGCG
miRNA  3' G   A               A         U         5'

------------------------------------------------------------

dataset: 1
target: OL672836.1
length: 29684
miRNA : 5008c
length: 30

mfe: -32.0 kcal/mol
p-value: 1.000000e+00

position  4625
target 5' C      CAGUUUC   UUCU     UG      GUUA      A 3'
           CAGCUA       UGU    UCACC   AUGCU    CAGCGU
           GUCGAU       ACG    AGUGG   UAUGG    GUUGCG
miRNA  3'                           UGA     UG          5'

------------------------------------------------------------

dataset: 1
target: OL672836.1
length: 29684
miRNA : 5008c
length: 30

mfe: -32.0 kcal/mol
p-value: 1.000000e+00
```

```
position  26725
target 5' U      CUUCAU        UUCAG    U UG   GU    G 3'
           CAGCUA      UGCUU CU     ACUG U  CGC  ACGC
           GUCGAU      ACGAG GG     UGAU A  GUG  UGCG
miRNA  3'                   U             UG   GU      5'

------------------------------------------------------------

dataset: 1
target: OL672836.1
length: 29684
miRNA : 5008c
length: 30

mfe: -31.7 kcal/mol
p-value: 1.000000e+00

position  16861
target 5' A    A    CAUUAAGUG    UAC    G      AAGA  A 3'
           CAGU AUGC         CACC   ACUA UGCCAC    GC
           GUCG UACG         GUGG   UGAU AUGGUG    CG
miRNA  3'      A    A                          GUUG    5'

------------------------------------------------------------

dataset: 1
target: OL672836.1
length: 29684
miRNA : 5008c
length: 30

mfe: -31.7 kcal/mol
p-value: 1.000000e+00

position  4125
target 5' A        GG        AAA        A 3'
           GGCUG GU  CACUACUG   UGCUAGCG
           UCGAU CG  GUGGUGAU   GUGGUUGC
miRNA  3' G     A  A         AUG        G 5'

------------------------------------------------------------

dataset: 1
target: OL672836.1
length: 29684
miRNA : 5008c
length: 30

mfe: -31.6 kcal/mol
p-value: 1.000000e+00

position  1263
target 5' A  AAG            UGUGGUUACU        AA    U 3'
           AG   GUGC CACUACU          UACC CCA  AUGC
           UC   UACG GUGGUGA          AUGG GGU  UGCG
miRNA  3' G  GA     A       U             U           5'
```

```
------------------------------------------------------------

dataset: 1
target: OL672836.1
length: 29684
miRNA : 5008c
length: 30

mfe: -31.6 kcal/mol
p-value: 1.000000e+00

position  29090
target 5' U   C    AAA UG   AAUU    C       U 3'
           GGC   GC   U  CAC    UGCC CCAGCGC
           UCG   CG   G  GUG    AUGG GGUUGCG
miRNA  3' G   AUA  A   UG   AU      U         5'

------------------------------------------------------------

dataset: 1
target: OL672836.1
length: 29684
miRNA : 5008c
length: 30

mfe: -31.5 kcal/mol
p-value: 1.000000e+00

position  26919
target 5'  C    UGACAUCAAGGACC   CUAAAGAAA     GUU    C     G     U 3'
            GCUG              UGC         UCACU   GCUA AUCAC AACGC
            CGAU              ACG         AGUGG   UGAU UGGUG UUGCG
miRNA  3' GU                                          A     G       5'

------------------------------------------------------------

dataset: 1
target: OL672836.1
length: 29684
miRNA : 5008c
length: 30

mfe: -31.5 kcal/mol
p-value: 1.000000e+00

position  14723
target 5' U    G  UA G        CGUUAUAAU        A  3'
           CAGC AU  U ACUACUAU         CUACCAAC
           GUCG UA  A UGGUGAUA         GGUGGUUG
miRNA  3'      A  CG G        U                CG 5'

------------------------------------------------------------

dataset: 1
target: OL672836.1
length: 29684
miRNA : 5008c
```

length: 30

mfe: -31.4 kcal/mol
p-value: 1.000000e+00

position 16139
```
target 5' A        ACA           AGU  UAC G  U 3'
           GGCUAUGU   CACCGC AUAC   CU   A GC
           UCGAUACG   GUGGUG UAUG   GG   U CG
miRNA  3' G        A        A    GU   U   G    5'
```

----

dataset: 1
target: OL672836.1
length: 29684
miRNA : 5008c
length: 30

mfe: -31.4 kcal/mol
p-value: 1.000000e+00

position 29632
```
target 5'       A         AUUU       G  C 3'
                 GC CACCAC    UCACCGA GC
                 CG GUGGUG    GGUGGUU CG
miRNA  3' GUCGAUA  A      AUAU       G    5'
```

----

dataset: 1
target: OL672836.1
length: 29684
miRNA : 5008c
length: 30

mfe: -31.3 kcal/mol
p-value: 1.000000e+00

position 11050
```
target 5'  U      GGUA           U     UU   A 3'
            GCUAUG    UUAUUGCUAUG CUGCU  UGC
            CGAUAC    AGUGGUGAUAU GGUGG  GCG
miRNA  3' GU      G                    UU     5'
```

----

dataset: 1
target: OL672836.1
length: 29684
miRNA : 5008c
length: 30

mfe: -31.2 kcal/mol
p-value: 1.000000e+00

position 13162

```
target 5' U   G    A   UGU GU           U   C 3'
           GGU GUGC UCG   U  CUGUACUGCCG UGC
           UCG UACG AGU   G  GAUAUGGUGGU GCG
miRNA  3' G   A            GU           U     5'
```

```
dataset: 1
target: OL672836.1
length: 29684
miRNA : 5008c
length: 30

mfe: -31.1 kcal/mol
p-value: 1.000000e+00

position  1124
target 5' C        G      AAUGA    A     AUG   C 3'
           CAGU  UGC UCACCA     AUGC ACCAA   UGC
           GUCG  ACG AGUGGU     UAUG UGGUU   GCG
miRNA  3'     AU          GA       G             5'
```

```
dataset: 1
target: OL672836.1
length: 29684
miRNA : 5008c
length: 30

mfe: -31.1 kcal/mol
p-value: 1.000000e+00

position  2811
target 5' A   AAA  A       UG   UG  G  G    U 3'
           AGU   UG GUUCGCC  UGU  UG CA AUGC
           UCG   AU CGAGUGG  AUA  GU GU UGCG
miRNA  3' G        A       UG   UG  G         5'
```

```
dataset: 1
target: OL672836.1
length: 29684
miRNA : 5008c
length: 30

mfe: -30.9 kcal/mol
p-value: 1.000000e+00

position  18167
target 5' A    A   G         ACAUGUU       C   A 3'
           AGUU AUG UUACC CUA       UAUCACC GCG
           UCGA UAC AGUGG GAU       AUGGUGG UGC
miRNA  3' G        G     U                 U   G 5'
```

```
dataset: 1
target: OL672836.1
length: 29684
miRNA : 5008c
length: 30

mfe: -30.9 kcal/mol
p-value: 1.000000e+00

position  25178
target 5' A  A        UG UG   G    G      U 3'
           CA UUAUGCUU  C  UAU ACCA U  UGC
           GU GAUACGAG  G  AUA UGGU G  GCG
miRNA  3'    C        UG UG        G UU     5'

------------------------------------------------------------
```

BA.2 top 25 for tDR-Val

```
Version: RNAhybrid 2.2
Command line:/vol/bioapps/bin/RNAhybrid.bin -t
/var/bibiserv2/anonymous/rnahybrid/28/15/12/bibiserv2_2022-06-
28_151239_34y4j/rnahybrid_input_target_rna_sequences_.file -m 29780 -n 31 -b
25 -s 3utr_human -q
/var/bibiserv2/anonymous/rnahybrid/28/15/12/bibiserv2_2022-06-
28_151239_34y4j/rnahybrid_input_mirna_sequences.file
searching
dataset: 1
mde of 5027: -64.200005
Individual hits
------------------------------------------------------------

dataset: 1
target: ON024493.1
length: 29780
miRNA : 5027
length: 31

mfe: -31.0 kcal/mol
p-value: 1.000000e+00

position  6133
target 5' G                  GAUUAUAA       A      3'
           GUG AUGUGGUGGCUAUU        ACACUAC
           CGC UGCACUAUUGGUGA        UGUGAUG
miRNA  3'     U                             CCUUUG 5'

------------------------------------------------------------

dataset: 1
target: ON024493.1
length: 29780
```

```
miRNA : 5027
length: 31

mfe: -30.5 kcal/mol
p-value: 1.000000e+00

position  5482
target 5' U           UGUAAA   UG  G  A  A C    A 3'
           UGAACGUGGUG      ACU  UG AC AC G AGAC
           GCUUGCACUAU      UGG  AU UG UG C UUUG
miRNA  3' C                    UG  G  A  C        5'

------------------------------------------------------------

dataset: 1
target: ON024493.1
length: 29780
miRNA : 5027
length: 31

mfe: -30.0 kcal/mol
p-value: 1.000000e+00

position  6428
target 5' A   U      A      CGAAG UG        A 3'
           GUG AAUGUGA AACUAC     U  UA GGAGAC
           CGC UUGCACU UUGGUG     G  AU CCUUUG
miRNA  3'            A      AU    UG  G        5'

------------------------------------------------------------

dataset: 1
target: ON024493.1
length: 29780
miRNA : 5027
length: 31

mfe: -29.9 kcal/mol
p-value: 1.000000e+00

position  29210
target 5' C G        U            A    U   C 3'
           G GAACGUGG UGACC  UACAC   GG  GC
           C CUUGCACU AUUGG  AUGUG   CC  UG
miRNA  3'   G             UG     AUG  UU    5'

------------------------------------------------------------

dataset: 1
target: ON024493.1
length: 29780
miRNA : 5027
length: 31

mfe: -29.6 kcal/mol
p-value: 1.000000e+00
```

```
position  12203
target 5' U   C       CA     GCA  GUAAGU      A 3'
           UGA CGUGAUG  GCCAU   AC      UGGAAA
           GCU GCACUAU  UGGUG   UG      GCCUUU
miRNA  3' C   U              AUG  AU          G 5'

------------------------------------------------------------

dataset: 1
target: ON024493.1
length: 29780
miRNA : 5027
length: 31

mfe: -29.3 kcal/mol
p-value: 1.000000e+00

position  10092
target 5'     U       CA         UA    UCUUU   U 3'
               UGUGGUA   ACUACACU  ACGG     GGC
               GCACUAU   UGAUGUGA  UGCC     UUG
miRNA  3' CGCUU       UGG              U         5'

------------------------------------------------------------

dataset: 1
target: ON024493.1
length: 29780
miRNA : 5027
length: 31

mfe: -29.2 kcal/mol
p-value: 1.000000e+00

position  10643
target 5'   U   U    UGUA      UG UAUAA        A 3'
             AGC UGGU    CGCUGC  U     AUGGAGAC
             UUG ACUA    GUGAUG  A     UGCCUUUG
miRNA  3' CGC   C    UUG       UG              5'

------------------------------------------------------------

dataset: 1
target: ON024493.1
length: 29780
miRNA : 5027
length: 31

mfe: -29.0 kcal/mol
p-value: 1.000000e+00

position  1432
target 5' C  A  G     CGC           UU      U 3'
           GU AG GUGGU   ACUAUUGC CU  GGAGGC
           CG UU CACUA   UGGUGAUG GA  CCUUUG
miRNA  3'    C  G     U          U  UG        5'
```

```
------------------------------------------------------------

dataset: 1
target: ON024493.1
length: 29780
miRNA : 5027
length: 31

mfe: -29.0 kcal/mol
p-value: 1.000000e+00

position  8755
target 5' A  C        GG AG        AU  CAA   U 3'
           GC AGCGUGGU  U  UUAUACUA  GA   AGC
           CG UUGCACUA  G  GAUGUGAU  CU   UUG
miRNA  3'    C        UU GU        GC          5'

------------------------------------------------------------

dataset: 1
target: ON024493.1
length: 29780
miRNA : 5027
length: 31

mfe: -29.0 kcal/mol
p-value: 1.000000e+00

position  29053
target 5' G  A             GA   AACCCAA      U 3'
           GC GACGUGGU  CCA  ACA       GGAAAU
           CG UUGCACUA  GGU  UGU       CCUUUG
miRNA  3'    C        UU   GA   GAUG           5'

------------------------------------------------------------

dataset: 1
target: ON024493.1
length: 29780
miRNA : 5027
length: 31

mfe: -28.8 kcal/mol
p-value: 1.000000e+00

position  628
target 5'  G               A   G     C    3'
            GAGC UGGUGGCCAU   G UACGG
            CUUG ACUAUUGGUG   U AUGCC
miRNA  3' CG    C          AUG G     UUUG 5'

------------------------------------------------------------

dataset: 1
target: ON024493.1
length: 29780
miRNA : 5027
```

```
length: 31

mfe: -28.7 kcal/mol
p-value: 1.000000e+00

position  6341
target 5' A G                        A    C 3'
           G GAAUG GAUAAUC UUGC CUGCG AGAU
           C CUUGC CUAUUGG GAUG GAUGC UUUG
miRNA  3'   G     A       U    U     C      5'

------------------------------------------------------------

dataset: 1
target: ON024493.1
length: 29780
miRNA : 5027
length: 31

mfe: -28.6 kcal/mol
p-value: 1.000000e+00

position  4133
target 5'     C      UAU   U   AAAAAGGCUAGUG       U     G 3'
               UGUGGU   ACC ACU             GCACUAC GAAAU
               GCACUA   UGG UGA             UGUGAUG CUUUG
miRNA  3' CGCUU      U                             C       5'

------------------------------------------------------------

dataset: 1
target: ON024493.1
length: 29780
miRNA : 5027
length: 31

mfe: -28.4 kcal/mol
p-value: 1.000000e+00

position  27030
target 5' C         UAGCAG      CAGGUUUU     AUACAGUC     A   UU   A 3'
           GC AGCGUG      GUGACU        GCUGC        GCUAC GGA  GGC
           CG UUGCAC      UAUUGG        UGAUG        UGAUG CCU  UUG
miRNA  3'    C                                                     5'

------------------------------------------------------------

dataset: 1
target: ON024493.1
length: 29780
miRNA : 5027
length: 31

mfe: -28.0 kcal/mol
p-value: 1.000000e+00

position  2221
```

```
target 5' G      AAAU  UC    UG    AA        U 3'
           GUGGAC    UG   ACC  UGCA    GGAAAU
           CGCUUG    AC   UGG  AUGU    CCUUUG
miRNA  3'        C     UAU   UG    GAUG        5'

------------------------------------------------------------

dataset: 1
target: ON024493.1
length: 29780
miRNA : 5027
length: 31

mfe: -27.8 kcal/mol
p-value: 1.000000e+00

position  8675
target 5' U   UG            CGUGACAUA   U    A  U  U 3'
           UGA  GUGGUG UCACU         GCA CUAC GA AC
           GCU  CACUAU GGUGA         UGU GAUG CU UG
miRNA  3' C   UG      U                      C  U    5'

------------------------------------------------------------

dataset: 1
target: ON024493.1
length: 29780
miRNA : 5027
length: 31

mfe: -27.7 kcal/mol
p-value: 1.000000e+00

position  28549
target 5'  A  UG     UU                C  3'
            GA  GU AU  CUACUAC CUA GGAA
            CU  CA UA  GGUGAUG GAU CCUU
miRNA  3' CG  UG  C  UU       U   G    UG 5'

------------------------------------------------------------

dataset: 1
target: ON024493.1
length: 29780
miRNA : 5027
length: 31

mfe: -27.7 kcal/mol
p-value: 1.000000e+00

position  17123
target 5' A  UU  UCUCAU      GUUGAU        U    A 3'
           GC  GC      GCCGCU      GCACUAUG GAGA
           CG  UG      UGGUGA      UGUGAUGC CUUU
miRNA  3'    CU  CACUAU                         G 5'

------------------------------------------------------------
```

dataset: 1
target: ON024493.1
length: 29780
miRNA : 5027
length: 31

mfe: -27.7 kcal/mol
p-value: 1.000000e+00

position 19809
```
target 5' U  GG     ACAUU      A      UGAUC     CU  A 3'
           UG  UGUGG     GCUGCU AUACUG     UGGGA  AC
           GC  GCACU     UGGUGA UGUGAU     GCCUU  UG
miRNA  3' C  UU     AU                                5'
```

----

dataset: 1
target: ON024493.1
length: 29780
miRNA : 5027
length: 31

mfe: -27.7 kcal/mol
p-value: 1.000000e+00

position 751
```
target 5' C    ACUCAU      GCU    GGAGGGGCAUA            UC   A 3'
           GUGA      GCGUGA   UAAC           CACU CGCUAUG  GAU
           CGCU      UGCACU   AUUG           GUGA GUGAUGC  UUG
miRNA  3'                                        U       CU     5'
```

----

dataset: 1
target: ON024493.1
length: 29780
miRNA : 5027
length: 31

mfe: -27.6 kcal/mol
p-value: 1.000000e+00

position 25888
```
target 5' C     A   CU    GAUUGGUGG       GAAAA        C 3'
           UGAAC UGA  ACCA         UUAUACU     AUGGGAAU
           GCUUG ACU  UGGU         GAUGUGA     UGCCUUUG
miRNA  3' C     C   AU                                   5'
```

----

dataset: 1
target: ON024493.1
length: 29780
miRNA : 5027
length: 31

```
mfe: -27.4 kcal/mol
p-value: 1.000000e+00

position  3494
target 5' U     C          AUA     UAAU   C   3'
           UGAAU UGAUGAUUAC   GCUAC    GGA
           GCUUG ACUAUUGGUG   UGAUG    CCU
miRNA  3' C     C          AUG            UUG 5'

------------------------------------------------------------

dataset: 1
target: ON024493.1
length: 29780
miRNA : 5027
length: 31

mfe: -27.4 kcal/mol
p-value: 1.000000e+00

position  29661
target 5'  A    U   AAG      CACAUUUUCACC   AGUGAACAAU    G     G 3'
            GGAC UGA   AGCCAC            UAC          GCUA GGAGA
            CUUG ACU   UUGGUG            AUG          UGAU CCUUU
miRNA  3' CG    C   A                                     G     G 5'

------------------------------------------------------------

dataset: 1
target: ON024493.1
length: 29780
miRNA : 5027
length: 31

mfe: -27.4 kcal/mol
p-value: 1.000000e+00

position  4565
target 5' A    ACUCU   UACAAU      UG      U    A 3'
           UGAA     UGU      GCCACU  GCUAUG  AAC
           GCUU     GCA      UGGUGA  UGAUGC  UUG
miRNA  3' C            CUAU        UG      CU     5'

------------------------------------------------------------

dataset: 1
target: ON024493.1
length: 29780
miRNA : 5027
length: 31

mfe: -27.3 kcal/mol
p-value: 1.000000e+00

position  310
target 5' C         CUCG    GUG   UU      U 3'
```

```
           GCG ACGUG     UAC   GCU  GGAGAC
           CGC UGCAC     GUG   UGA  CCUUUG
miRNA  3'     U     UAUUG
AUG   UG        5'
```

Top 50 for BA.2 tDR-Gly

```
Version: RNAhybrid 2.2
Command line:/vol/bioapps/bin/RNAhybrid.bin -t
/var/bibiserv2/anonymous/rnahybrid/28/15/51/bibiserv2_2022-06-
28_155132_ofCoo/rnahybrid_input_target_rna_sequences_.file -s 3utr_human -b
50 -q /var/bibiserv2/anonymous/rnahybrid/28/15/51/bibiserv2_2022-06-
28_155132_ofCoo/rnahybrid_input_mirna_sequences.file -n 30 -m 29780
searching
dataset: 1
mde of 5008c: -65.000008
Individual hits
------------------------------------------------------------

dataset: 1
target: ON024493.1
length: 29780
miRNA : 5008c
length: 30

mfe: -35.7 kcal/mol
p-value: 1.000000e+00

position  1461
target 5' A      GUUCUCUUAUGUUGG      AACAAG     UAUUGGGUUCCACGU      U
3'
           GGCUGUGU              UUGCCAU     UGUGCC              GCUAGCGC
           UCGAUACG              AGUGGUG     AUAUGG              UGGUUGCG
miRNA  3' G
5'

------------------------------------------------------------

dataset: 1
target: ON024493.1
length: 29780
miRNA : 5008c
length: 30

mfe: -35.3 kcal/mol
p-value: 1.000000e+00

position  10372
target 5' U    UGUUACAAUG       U   GUGU         G 3'
           UAGCU         GUUCACCA CUG    UUACCAAUGU
           GUCGA         CGAGUGGU GAU    GGUGGUUGCG
miRNA  3'    UA                   AU             5'

------------------------------------------------------------

dataset: 1
```

```
target: ON024493.1
length: 29780
miRNA : 5008c
length: 30

mfe: -35.2 kcal/mol
p-value: 1.000000e+00

position  17121
target 5' A                         GUU     A 3'
           CAGCU UGCUC UCAU   GCCGCU   GAUGC
           GUCGA ACGAG GGUG   UGGUGG   UUGCG
miRNA  3'       U     U    AUA                5'

------------------------------------------------------------

dataset: 1
target: ON024493.1
length: 29780
miRNA : 5008c
length: 30

mfe: -33.0 kcal/mol
p-value: 1.000000e+00

position  14540
target 5'  U       A       UCUGGUAAUC        GAUAA    A 3'
            GCUAUGC CGCUGCU          UAUUACUA     ACGC
            CGAUACG GUGGUGA          AUGGUGGU     UGCG
miRNA  3' GU       A       U                            5'

------------------------------------------------------------

dataset: 1
target: ON024493.1
length: 29780
miRNA : 5008c
length: 30

mfe: -32.9 kcal/mol
p-value: 1.000000e+00

position  13726
target 5' A    GAC   G       A        GU      C 3'
           CGGU   AUG  UACCAC UAUAUCAC  CAACGU
           GUCG   UAC  GUGGUG AUAUGGUG  GUUGCG
miRNA  3'      A     GA                         5'

------------------------------------------------------------

dataset: 1
target: ON024493.1
length: 29780
miRNA : 5008c
length: 30

mfe: -32.7 kcal/mol
```

```
p-value: 1.000000e+00

position  17399
target 5' U   GACCC      AU        UG  CC    A 3'
           GGC     UGCUCA      UACC  CA  ACGC
           UCG     ACGAGU      AUGG  GU  UGCG
miRNA  3' G   AU         GGUGAU    UG          5'

------------------------------------------------------------

dataset: 1
target: ON024493.1
length: 29780
miRNA : 5008c
length: 30

mfe: -32.5 kcal/mol
p-value: 1.000000e+00

position  28377
target 5'  U  GUCUUG          CUC  U  A  UG  A 3'
            GC      GUUCACCGCU   AC CA CA  GC
            CG      CGAGUGGUGA   UG GU GU  CG
miRNA  3' GU  AUA             UA      G  UG    5'

------------------------------------------------------------

dataset: 1
target: ON024493.1
length: 29780
miRNA : 5008c
length: 30

mfe: -32.4 kcal/mol
p-value: 1.000000e+00

position  29463
target 5' G   C     G   CAACUCAGGCCUAA   CAUGCAG     A   G  A 3'
           AGC GUGCU ACU              ACU       ACCAC CAA GC
           UCG UACGA UGG              UGA       UGGUG GUU CG
miRNA  3' G   A     G                    UA              G    5'

------------------------------------------------------------

dataset: 1
target: ON024493.1
length: 29780
miRNA : 5008c
length: 30

mfe: -32.3 kcal/mol
p-value: 1.000000e+00

position  24183
target 5' U   GCAG     G      AA        UGCU    A 3'
           GGU    GUGCU CAUUAC  AUACCAUU    AUGC
           UCG    UACGA GUGGUG  UAUGGUGG    UGCG
```

```
miRNA  3' G   A               A         U         5'
```

----

dataset: 1
target: ON024493.1
length: 29780
miRNA : 5008c
length: 30

mfe: -32.0 kcal/mol
p-value: 1.000000e+00

```
position  4654
target 5' C      CAGUUUC   UUCU     UG      GUUA      A 3'
           CAGCUA       UGU    UCACC   AUGCU    CAGCGU
           GUCGAU       ACG    AGUGG   UAUGG    GUUCGG
miRNA  3'                           UGA     UG          5'
```

----

dataset: 1
target: ON024493.1
length: 29780
miRNA : 5008c
length: 30

mfe: -32.0 kcal/mol
p-value: 1.000000e+00

```
position  4154
target 5' A        GG        AAA        A 3'
           GGCUA GU  CACUACUG   UGCUAGCG
           UCGAU CG  GUGGUGAU   GUGGUUGC
miRNA  3' G     A  A         AUG        G 5'
```

----

dataset: 1
target: ON024493.1
length: 29780
miRNA : 5008c
length: 30

mfe: -32.0 kcal/mol
p-value: 1.000000e+00

```
position  26757
target 5' U      CUUCAU        UUCAG    U UG   GU    G 3'
           CAGCUA      UGCUU CU     ACUG U  CGC  ACGC
           GUCGAU      ACGAG GG     UGAU A  GUG  UGCG
miRNA  3'                   U             UG   GU      5'
```

----

dataset: 1
target: ON024493.1

```
length: 29780
miRNA : 5008c
length: 30

mfe: -31.7 kcal/mol
p-value: 1.000000e+00

position  16893
target 5' A    A    CAUUAAGUG    UAC    G      AAGA  A 3'
           CAGU AUGC         CACC   ACUA UGCCAC    GC
           GUCG UACG         GUGG   UGAU AUGGUG    CG
miRNA  3'      A    A                          GUUG    5'

------------------------------------------------------------

dataset: 1
target: ON024493.1
length: 29780
miRNA : 5008c
length: 30

mfe: -31.6 kcal/mol
p-value: 1.000000e+00

position  1292
target 5' A  AAG            UGUGGUUACU        AA    U 3'
           AG   GUGC CACUACU          UACC CCA   AUGC
           UC   UACG GUGGUGA          AUGG GGU   UGCG
miRNA  3' G  GA     A       U             U           5'

------------------------------------------------------------

dataset: 1
target: ON024493.1
length: 29780
miRNA : 5008c
length: 30

mfe: -31.6 kcal/mol
p-value: 1.000000e+00

position  29131
target 5' U   C    AAA UG   AAUU    C       U 3'
           GGC   GC   U  CAC    UGCC CCAGCGC
           UCG   CG   G  GUG    AUGG GGUUGCG
miRNA  3' G   AUA  A   UG   AU      U         5'

------------------------------------------------------------

dataset: 1
target: ON024493.1
length: 29780
miRNA : 5008c
length: 30

mfe: -31.5 kcal/mol
p-value: 1.000000e+00
```

```
position  26951
target 5'  C    UGACAUCAAGGACC   CUAAAGAAA     GUU    C     G     U 3'
            GCUG              UGC         UCACU   GCUA AUCAC AACGC
            CGAU              ACG         AGUGG   UGAU UGGUG UUGCG
miRNA  3' GU                                          A     G       5'
```

----

dataset: 1
target: ON024493.1
length: 29780
miRNA : 5008c
length: 30

mfe: -31.5 kcal/mol
p-value: 1.000000e+00

```
position  14755
target 5' U    G  UA G        CGUUAUAAU        A  3'
           CAGC AU  U ACUACUAU         CUACCAAC
           GUCG UA  A UGGUGAUA         GGUGGUUG
miRNA  3'      A  CG G        U                CG 5'
```

----

dataset: 1
target: ON024493.1
length: 29780
miRNA : 5008c
length: 30

mfe: -31.4 kcal/mol
p-value: 1.000000e+00

```
position  16171
target 5' A        ACA           AGU  UAC G  U 3'
           GGCUAUGU   CACCGC AUAC   CU   A GC
           UCGAUACG   GUGGUG UAUG   GG   U CG
miRNA  3' G        A        A    GU   U   G    5'
```

----

dataset: 1
target: ON024493.1
length: 29780
miRNA : 5008c
length: 30

mfe: -31.3 kcal/mol
p-value: 1.000000e+00

```
position  11082
target 5'  U      GGUA           U     UU   A 3'
            GCUAUG    UUAUUGCUAUG CUGCU  UGC
            CGAUAC    AGUGGUGAUAU GGUGG  GCG
miRNA  3' GU      G                    UU     5'
```

```
----------------------------------------------------------------

dataset: 1
target: ON024493.1
length: 29780
miRNA : 5008c
length: 30

mfe: -31.2 kcal/mol
p-value: 1.000000e+00

position  13194
target 5' U   G    A   UGU GU           U   C 3'
           GGU GUGC UCG   U  CUGUACUGCCG UGC
           UCG UACG AGU   G  GAUAUGGUGGU GCG
miRNA  3' G   A            GU           U     5'

----------------------------------------------------------------

dataset: 1
target: ON024493.1
length: 29780
miRNA : 5008c
length: 30

mfe: -31.1 kcal/mol
p-value: 1.000000e+00

position  1153
target 5' C         G      AAUGA    A     AUG   C 3'
           CAGU  UGC UCACCA     AUGC ACCAA   UGC
           GUCG  ACG AGUGGU     UAUG UGGUU   GCG
miRNA  3'      AU          GA       G             5'

----------------------------------------------------------------

dataset: 1
target: ON024493.1
length: 29780
miRNA : 5008c
length: 30

mfe: -31.1 kcal/mol
p-value: 1.000000e+00

position  2840
target 5' A   AAA  A       UG   UG  G  G    U 3'
           AGU   UG GUUCGCC  UGU  UG CA AUGC
           UCG   AU CGAGUGG  AUA  GU GU UGCG
miRNA  3' G        A       UG   UG  G         5'

----------------------------------------------------------------

dataset: 1
target: ON024493.1
length: 29780
```

```
miRNA : 5008c
length: 30

mfe: -30.9 kcal/mol
p-value: 1.000000e+00

position  18199
target 5' A    A   G         ACAUGUU       C   A 3'
           AGUU AUG UUACC CUA       UAUCACC GCG
           UCGA UAC AGUGG GAU       AUGGUGG UGC
miRNA  3' G        G     U                 U   G 5'

------------------------------------------------------------

dataset: 1
target: ON024493.1
length: 29780
miRNA : 5008c
length: 30

mfe: -30.9 kcal/mol
p-value: 1.000000e+00

position  25210
target 5' A  A        UG UG   G    G      U 3'
           CA UUAUGCUU  C  UAU ACCA U  UGC
           GU GAUACGAG  G  AUA UGGU G  GCG
miRNA  3'    C        UG UG        G UU     5'

------------------------------------------------------------

dataset: 1
target: ON024493.1
length: 29780
miRNA : 5008c
length: 30

mfe: -30.9 kcal/mol
p-value: 1.000000e+00

position  13037
target 5'   U      U  UG UG  GA         A  U 3'
             CUGUGC UU  C  UA  UGCUGCUAA GC
             GAUACG AG  G  AU  AUGGUGGUU CG
miRNA  3' GUC         UG UG             G    5'

------------------------------------------------------------

dataset: 1
target: ON024493.1
length: 29780
miRNA : 5008c
length: 30

mfe: -30.7 kcal/mol
p-value: 1.000000e+00
```

```
position  15990
target 5' U      UAGA        C        AAACAUCCUA    GGAGU    U 3'
           UAGCUA    UGCUUACC ACU UACU          AUCA     AUGC
           GUCGAU    ACGAGUGG UGA AUGG          UGGU     UGCG
miRNA  3'                        U                             5'
```

```
dataset: 1
target: ON024493.1
length: 29780
miRNA : 5008c
length: 30

mfe: -30.6 kcal/mol
p-value: 1.000000e+00

position  13373
target 5' G        A   ACU    GAACCC     UCAGUCA        A 3'
           UAGUUGUG UCA   CCGC      AUGCU       GCUGAUGC
           GUCGAUAC AGU   GGUG      UAUGG       UGGUUGCG
miRNA  3'          G          A                         5'
```

```
dataset: 1
target: ON024493.1
length: 29780
miRNA : 5008c
length: 30

mfe: -30.6 kcal/mol
p-value: 1.000000e+00

position  12393
target 5' U          CCUUGAA   A     UCUUA    A  A 3'
           GGUUGUGUUC       CAU AUACC     CAAC GC
           UCGAUACGAG       GUG UAUGG     GUUG CG
miRNA  3' G          UG        A     UG            5'
```

```
dataset: 1
target: ON024493.1
length: 29780
miRNA : 5008c
length: 30

mfe: -30.6 kcal/mol
p-value: 1.000000e+00

position  16118
target 5'  U             AUG   A   UU   G  A 3'
            GUUAUGCUUACUA   AUA CAC  CAA GU
            CGAUACGAGUGGU   UAU GUG  GUU CG
miRNA  3' GU             GA    G        G    5'
```

```
------------------------------------------------------------

dataset: 1
target: ON024493.1
length: 29780
miRNA : 5008c
length: 30

mfe: -30.6 kcal/mol
p-value: 1.000000e+00

position  26654
target 5' C    AACUUUAACUUGUUU               UU  AGAAUAAAUUGGAU    GG  G 3'
           CAGU               UGUGCUUGCUGCUGU  AC              CACC  UG
           GUCG               AUACGAGUGGUGAUA  UG              GUGG  GC
miRNA  3'                                                          UU  G 5'

------------------------------------------------------------

dataset: 1
target: ON024493.1
length: 29780
miRNA : 5008c
length: 30

mfe: -30.3 kcal/mol
p-value: 1.000000e+00

position  12318
target 5' G   A A      U         UAGAAAGUUGGAUAA      A 3'
           CAG C AUGCUU UCACUAUGCU               UGAUGC
           GUC G UACGAG GGUGAUAUGG               GUUGCG
miRNA  3'      A      U          UG                    5'

------------------------------------------------------------

dataset: 1
target: ON024493.1
length: 29780
miRNA : 5008c
length: 30

mfe: -30.3 kcal/mol
p-value: 1.000000e+00

position  1986
target 5'  U    UGA       AU   AUUUG         A  3'
            GCUA   UGUUCAC  CUG     GCUACUAAC
            CGAU   ACGAGUG  GAU     UGGUGGUUG
miRNA  3' GU              GU   A             CG 5'

------------------------------------------------------------

dataset: 1
target: ON024493.1
length: 29780
miRNA : 5008c
```

length: 30

mfe: -30.2 kcal/mol
p-value: 1.000000e+00

position 15075
```
target 5' G      G  U   U   UG A     AUG      A  3'
           UAGCUG UG CUC AUC  U GUACU   ACCAAU
           GUCGAU AC GAG UGG  A UAUGG   UGGUUG
miRNA  3'                   UG                CG 5'
```

----

dataset: 1
target: ON024493.1
length: 29780
miRNA : 5008c
length: 30

mfe: -30.0 kcal/mol
p-value: 1.000000e+00

position 4575
```
target 5'  U    CAA        UG      UA  A  UG  U 3'
            GUUA   UGC CACU  GCUAUG  AC CA  GC
            CGAU   ACG GUGG  UGAUAU  UG GU  CG
miRNA  3' GU          A            GG     UG    5'
```

----

dataset: 1
target: ON024493.1
length: 29780
miRNA : 5008c
length: 30

mfe: -30.0 kcal/mol
p-value: 1.000000e+00

position 11400
```
target 5' U   A      UAGAUCAA      U      GU G  U 3'
           GGU AUGCUU        GCCAUU   CCAU  G GC
           UCG UACGAG        UGGUGA   GGUG  U CG
miRNA  3' G   A                    UAU    GU G    5'
```

----

dataset: 1
target: ON024493.1
length: 29780
miRNA : 5008c
length: 30

mfe: -29.9 kcal/mol
p-value: 1.000000e+00

position 9326

```
target 5'    A      UA        AUUCA   U   GG   U 3'
              UAUGUU  CACCACUA     ACC AUU  UGC
              AUACGA  GUGGUGAU     UGG UGG  GCG
miRNA  3' GUCG                A           UU     5'

------------------------------------------------------------

dataset: 1
target: ON024493.1
length: 29780
miRNA : 5008c
length: 30

mfe: -29.9 kcal/mol
p-value: 1.000000e+00

position  435
target 5' A    CCUA           AACGUUCGG       G   U  A 3'
           CAGC    UGUGUUCAUCA         AUGCU C AAC GC
           GUCG    AUACGAGUGGU         UAUGG G UUG CG
miRNA  3'                     GA            U G        5'

------------------------------------------------------------

dataset: 1
target: ON024493.1
length: 29780
miRNA : 5008c
length: 30

mfe: -29.8 kcal/mol
p-value: 1.000000e+00

position  15435
target 5' C   G  GAAC       GGAG           U  U 3'
           CAG UG    CUCAUCA    AUGCCAC AAC GC
           GUC AU    GAGUGGU    UAUGGUG UUG CG
miRNA  3'     G  AC         GA         G        5'

------------------------------------------------------------

dataset: 1
target: ON024493.1
length: 29780
miRNA : 5008c
length: 30

mfe: -29.8 kcal/mol
p-value: 1.000000e+00

position  24079
target 5' A    UAACG C         U       UU   U 3'
           AGUU     G CUUACUGUU UGCCACC  UGC
           UCGA     C GAGUGGUGA AUGGUGG  GCG
miRNA  3' G    UA              U       UU     5'

------------------------------------------------------------
```

```
dataset: 1
target: ON024493.1
length: 29780
miRNA : 5008c
length: 30

mfe: -29.7 kcal/mol
p-value: 1.000000e+00

position  23578
target 5' G      GUCAAUCCAUCAU   CUA            U GG   A 3'
           UAGCUA             UGC    CACUAUGUCAC U  UGC
           GUCGAU             ACG    GUGAUAUGGUG G  GCG
miRNA  3'                        AGUG             UU     5'

------------------------------------------------------------

dataset: 1
target: ON024493.1
length: 29780
miRNA : 5008c
length: 30

mfe: -29.7 kcal/mol
p-value: 1.000000e+00

position  26281
target 5'  U        A            U  UUACU    U 3'
            GCUA GUU   CACUA GCCA CC     GCGC
            CGAU CGA   GUGAU UGGU GG     UGCG
miRNA  3' GU    A   GUG     A       U          5'

------------------------------------------------------------

dataset: 1
target: ON024493.1
length: 29780
miRNA : 5008c
length: 30

mfe: -29.7 kcal/mol
p-value: 1.000000e+00

position  64
target 5' U      CA   G          UUAGUG    C    A 3'
           GGCUGU  CUC GCUGC AUGC      CACU ACGC
           UCGAUA  GAG UGGUG UAUG      GUGG UGCG
miRNA  3' G      C          A              U      5'

------------------------------------------------------------

dataset: 1
target: ON024493.1
length: 29780
miRNA : 5008c
length: 30
```

```
mfe: -29.6 kcal/mol
p-value: 1.000000e+00

position  26121
target 5' C    AAUGGAACCAAUU    AUGA   G  GACG           G 3'
           CAGU             UAUG    ACC AC    ACUACUAGCGU
           GUCG             AUAC    UGG UG    UGGUGGUUGCG
miRNA  3'                       GAG       AUA              5'

------------------------------------------------------------

dataset: 1
target: ON024493.1
length: 29780
miRNA : 5008c
length: 30

mfe: -29.6 kcal/mol
p-value: 1.000000e+00

position  18814
target 5' C        AUGCAA    G    GGUGUCUAGCUGU    G     U 3'
           UAGUUGUG      UCAU ACUA             CCAC AGUGC
           GUCGAUAC      AGUG UGAU             GGUG UUGCG
miRNA  3'          G         G    AU               G       5'

------------------------------------------------------------

dataset: 1
target: ON024493.1
length: 29780
miRNA : 5008c
length: 30

mfe: -29.5 kcal/mol
p-value: 1.000000e+00

position  3688
target 5' U     GGUAUUUUUG     G          AUU  UUAAGA  U 3'
           CAGCU          GUGCU ACC CUAUAC   CU      GU
           GUCGA          UACGA UGG GAUAUG   GG      CG
miRNA  3'                      G   U      GU   UUG       5'

------------------------------------------------------------

dataset: 1
target: ON024493.1
length: 29780
miRNA : 5008c
length: 30

mfe: -29.4 kcal/mol
p-value: 1.000000e+00

position  9626
target 5' U            A    UUA     UUUCUGGAUAA   U   U 3'
```

```
           GGUUAUGUUCAC C CU   GUACC           CAA UGC
           UCGAUACGAGUG G GA   UAUGG           GUU GCG
miRNA  3' G              U          UG                  5'

------------------------------------------------------------

dataset: 1
target: ON024493.1
length: 29780
miRNA : 5008c
length: 30

mfe: -29.4 kcal/mol
p-value: 1.000000e+00

position  17075
target 5'  U     UGGCCUA    U      CCCU  UG U    A 3'
            GCUAU       GCUC CUACUA    UC  C  CGC
            CGAUA       CGAG GGUGAU    GG  G  GCG
miRNA  3' GU               U      AU    UG UU     5'

------------------------------------------------------------

dataset: 1
target: ON024493.1
length: 29780
miRNA : 5008c
length: 30

mfe: -29.4 kcal/mol
p-value: 1.000000e+00

position  2803
target 5' G  AA      UG        AGU G   U  3'
           AG  GUGCUC  C CUAUAC   U AAC
           UC  UACGAG  G GAUAUG   G UUG
miRNA  3' G  GA      UG U      GU  G   CG 5'

------------------------------------------------------------

dataset: 1
target: ON024493.1
length: 29780
miRNA : 5008c
length: 30

mfe: -29.4 kcal/mol
p-value: 1.000000e+00

position  10727
target 5' U       AAG   A     GA   U     A  3'
           GGCUAUG   UAC AUUAU  ACC CUAAC
           UCGAUAC   GUG UGAUA  UGG GGUUG
miRNA  3' G       GA    G          U     CG 5'

------------------------------------------------------------
```

dataset: 1
target: ON024493.1
length: 29780
miRNA : 5008c
length: 30

mfe: -29.3 kcal/mol
p-value: 1.000000e+00

position 12056

```
target 5' A      A C    GAGUUUAGUU     U        U    A 3'
           AGCUAU G CUCA          CC CU   CCAUCA AUGC
           UCGAUA C GAGU          GG GA   GGUGGU UGCG
miRNA  3' G                         U  UAU             5'
```
